# A universal workflow for creation, validation, and generalization of detailed neuronal models

**DOI:** 10.1016/j.patter.2023.100855

**Published:** 2023-10-04

**Authors:** Maria Reva, Christian Rössert, Alexis Arnaudon, Tanguy Damart, Darshan Mandge, Anıl Tuncel, Srikanth Ramaswamy, Henry Markram, Werner Van Geit

**Affiliations:** 1 Blue Brain Project, École polytechnique fédérale de Lausanne (EPFL), Campus Biotech, 1202 Geneva, Switzerland; 2 Laboratory of Neural Microcircuitry (LNMC), Brain Mind Institute, School of Life Sciences, École polytechnique fédérale de Lausanne (EPFL), 1015 Lausanne, Switzerland

## Abstract

Detailed single-neuron modeling is widely used to study neuronal functions. While cellular and functional diversity across the mammalian cortex is vast, most of the available computational tools focus on a limited set of specific features characteristic of a single neuron. Here, we present a generalized automated workflow for the creation of robust electrical models and illustrate its performance by building cell models for the rat somatosensory cortex. Each model is based on a 3D morphological reconstruction and a set of ionic mechanisms. We use an evolutionary algorithm to optimize neuronal parameters to match the electrophysiological features extracted from experimental data. Then we validate the optimized models against additional stimuli and assess their generalizability on a population of similar morphologies. Compared to the state-of-the-art canonical models, our models show 5-fold improved generalizability. This versatile approach can be used to build robust models of any neuronal type.

## Highlights

• We introduce a workflow that generates robust and detailed electrical models of neurons
• Models made with this workflow match features observed in experimental data
• These models show a 5-fold improvement in generalizability compared to existing ones
• This workflow provides invaluable tools for in-depth study of a variety of neuronal types

## The bigger picture

Unraveling the complexity of information processing within individual neurons remains a hard task within neuroscience. Our work builds upon the advancements made in computational modeling, introducing a highly versatile and automated workflow for the construction of detailed and robust neuronal models that accurately reproduce experimentally observed electrophysiological behaviors. Utilizing open-source tools, our approach integrates 3D morphological reconstructions of neurons with specific ionic mechanisms. The resulting models offer novel insights into the diversity of neuronal responses observed in the rat somatosensory cortex. Furthermore, these models exhibit a 5-fold enhancement in generalizability compared to canonical models currently in use. Our universal workflow functions as a powerful tool to guide future experiments and enhance our comprehension of neuronal information processing and biophysical mechanisms across various neuronal types.

## Teaser

This study presents an automated method for creating detailed models of neurons. The approach successfully captures the complexity of neuronal responses in the brain, specifically the rat somatosensory cortex, and improves generalizability upon existing models by a factor of five. This breakthrough provides a versatile tool for further neuroscience research, aiding in the understanding of how individual neurons work and allowing researchers to test hypotheses in a computational setting.

## Introduction

Biophysically detailed neuronal models enable *in silico* exploration of the underlying complexity of information processing in a single neuron. These models allow systematic and reversible manipulations of neuronal properties, which are not necessarily feasible in an experimental setup. Detailed neuronal models, therefore, provide valuable tools for hypothesis testing and to guide further experiments. Since these models incorporate neuronal morphology, they allow the study of fine elements of cell signaling in relation to the intricate details of neuronal biophysical mechanisms and morphological shape, unlike most point-based neuronal models (i.e., leaky integrate and fire^1^ and its variations, Izhikevich spiking neuron model,^2^ etc.). For example, such models helped to advance our understanding of the importance of morphology on neuronal excitability^3^^,^^4^^,^^5^ and the contribution of specific currents to cell function.^6^^,^^7^^,^^8^^,^^9^ In addition, they served as a basis to build neuronal circuits to simulate and study brain activity.^10^^,^^11^

In general, electrical models (e-models) are expected to reproduce experimentally observed electrophysiological behaviors. This can be quantified using a similarity score that can be computed directly as the difference between experimental and numerical traces (using metrics such as mean square distance or other measures of similarity between curves).^12^ Alternatively, model performance may be assessed by computing a score between features extracted from these traces. In an *ex vivo* setting, intrinsic neuronal properties are often characterized by the mean values of features extracted from recorded voltage traces. We therefore utilize the *Z* score to quantify the deviation of feature values extracted from the model from those obtained experimentally. Since neuronal model parameters such as ion channel conductances and passive membrane properties are currently not always experimentally measurable, obtaining a model with an accurate score requires either manual or automatic exploration of the parameter space.^13^^,^^14^^,^^15^ The latter can be achieved through stochastic global parameter optimization using evolutionary algorithms (EAs). Being simple to parallelize and effective in high dimensions, EAs have gained popularity in the field.^16^ In the current study, we use an indicator-based evolutionary algorithm (IBEA)^17^^,^^18^ with good performance according to benchmarks.^19^ While cell models are usually customized through feature extraction and parameter fitting for a specific study or released independently,^6^^,^^14^^,^^20^^,^^21^ to our knowledge only a few completely open-sourced and reproducible workflows of model optimization exist.^22^ Here, we present a fully integrated, single-cell model-building routine based on open-source tools.

Model building can serve to construct a cell model that would represent either a single biological cell or a predefined type of cells.^23^^,^^24^^,^^25^ Although the former approach is common,^22^ it has a few limitations. One such limitation is that, while building a neuronal model, it is preferable to restrict its parameters by a rich repertoire of measured neuronal behaviors. For example, for a model that includes a full morphological tree, it is beneficial not only to tune this model to somatic responses but also to take into account dendritic recordings. However, it is usually challenging to collect a battery of voltage recordings from several cellular compartments in the same neuron. This can be overcome by combining dendritic and somatic recordings that were acquired in different cells of the electrical type (e-type), as defined in the literature.^23^^,^^24^ Moreover, the task of building neuronal circuits requires constructing hundreds of thousands of neuronal models that represent the same neuronal type. Optimizing the parameters of such a large number of models is computationally prohibitive and time consuming. On the other hand, a canonical model requires only one run of optimizations allowing the study of properties of a neuronal type and can be used in large circuit building by applying the model to a set of morphologies that constitute the same morphological type.^11^

In this work, we developed a workflow for single model creation, which allows us to build canonical neuronal models. With this approach, we created 40 models representing 11 e-types in the juvenile rat somatosensory cortex (SSCx). For each cell type, we extracted a set of electrophysiological features that were used to optimize model parameters. Then, each canonical model was applied to a number of morphologies to assess its generalizability. Compared to the previous canonical e-models,^6^^,^^11^ the e-models presented in this study feature several enhancements. First, we propose a novel optimization routine that accounts for the patch-clamp experiment settings used to obtain single-cell recordings, specifically through the normalization of the injected current by the rheobase. Second, to capture a broad repertoire of cellular signaling, we have significantly diversified the cost function by incorporating new e-features and protocols. Third, we have included numerous biophysical mechanisms absent in previous e-models, such as improved intracellular calcium dynamics, sodium channel models, and the placement of more active channels in the dendrites. We also validated our models using protocols not utilized in the optimizations. To illustrate the advantage of the models created with our workflow, we compared our layer 5 thick-tufted pyramidal cell (L5PC) e-model with the state-of-the-art L5PC canonical e-model.^11^ The L5PC e-model created with our workflow shows improved generalizability in comparison with the other e-model, indicating that our e-model is more robust to minor alterations of the neuronal morphologies. In addition, using the layer 5 pyramidal cell (L5PC) e-model as a use case, we performed a dendritic e-model validation and an analysis of the optimized parameter space. We chose the L5PC e-models due to the fact that rodent L5PCs have been extensively studied and characterized in *ex vivo* conditions for over 30 years. Finally, we present a set of notebooks that allow the reader to follow all the steps of the workflow with the example of the L5PC.

## Results

### Single-cell model-building workflow

**Figure 1:**
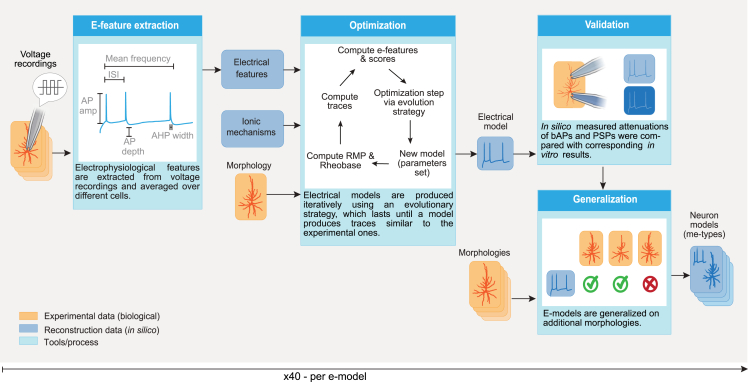
E-model-building workflow Schematic illustration of the steps involved in creating neuronal models. See “single-cell model-building workflow” section for a description of the pipeline.

Our goal was to develop an open-source workflow for generating robust e-models of neuronal cell types (Figure 1). Our workflow capitalizes on other open-source Python packages, such as BluePyEfe^26^ for the extraction of electrophysiological features, BluePyOpt^18^ to perform data-driven model optimization, and BluePyMM^27^ to generalize the electrical models to large sets of neuronal morphologies. The modular structure of our workflow with configuration files containing information about the model’s parameters, features, and cellular morphology allows for a flexible and multifaceted range of applications.

During the first step of the workflow, we extracted a set of electrophysiological features (e-features) from the voltage recordings of each cell that belongs to the same e-type using BluePyEfe (see sections “experimental procedures” and “electrophysiological features of the SSCx neuronal e-types”). The average values of the extracted features of all the cells of the same e-type were used as target values during the optimization process (Figure 1, E-feature extraction). The standard deviations were also computed and used as a way to normalize the e-feature scores during the optimization step.

The second step (Figure 1, optimization) aims at building canonical e-models that reproduce the previously obtained e-feature values when the neuron model is stimulated *in silico* with the same current stimulus. An e-model is composed of a detailed morphological reconstruction (exemplar morphology), mechanisms describing the dynamics of the passive and active electric properties of the membrane, and mechanisms describing the ionic dynamics inside the membrane of the cell (see Table S3). The free parameters of these mechanisms (e.g., maximum channel conductance, calcium decay) were optimized using an evolutionary algorithm^17^^,^^18^ with BluePyOpt. The cost function for the optimization is the sum of the errors between e-features produced by the e-model and the experimental e-features (Equation 1). The evolutionary algorithm then searches for a set of parameters that minimize this cost function. The second step (Figure 1, optimization) aims at building canonical e-models that reproduce the previously obtained e-feature values when the neuron model is stimulated *in silico* with the same current stimulus. An e-model is composed of a detailed morphological reconstruction (exemplar morphology), mechanisms describing the dynamics of the passive and active electric properties of the membrane, and mechanisms describing the ionic dynamics inside the membrane of the cell (see Table S3). The free parameters of these mechanisms (e.g., maximum channel conductance, calcium decay) were optimized using an evolutionary algorithm^17^^,^^18^ with BluePyOpt. The cost function for the optimization is the sum of the errors between e-features produced by the e-model and the experimental e-features (Equation 1). The evolutionary algorithm then searches for a set of parameters that minimize this cost function.

Once e-models were built, their quality was assessed by inspecting behaviors that were not specifically restricted with experimental data during the optimization (Figure 1, validation). This step checks for the reproduction of the attenuation of dendritic and synaptic potentials as well as somatic responses to the stimuli not used during optimizations (for details see section “validation and analysis of the detailed neuronal model”).

Finally, we tested morphological generalizability of the e-models using the BluePyMM software^27^). Since our models were optimized for a single morphology, we examined their performance on a broad collection of morphologies to select working pairs of morphologies and e-models, leading to what is referred to as morpho-electric models (me-models) (Figure 1). For example, this collection of *in silico* cells can be used to build a microcircuit^11^ (see sections “experimental procedures” and “generalization of electrical models”).

With our workflow, we generated e-models for the various firing and morphological neuronal types present in the juvenile rat SSCx. Based on the whole-cell patch-clamp single-cell recordings, each cell was manually assigned to one of 11 e-types,^23^ namely continuous adapting pyramidal cells (cADpyr) and 10 interneuron firing e-types: continuous accommodating (cAC), burst accommodating (bAC), continuous non-accommodating (cNAC), burst non-accommodating (bNAC), delayed non-accommodating (dNAC), delayed stuttering (dSTUT), burst irregular firing (bIR), continuous irregular firing (cIR), burst stuttering (bSTUT), and continuous stuttering (cSTUT). One e-model was generated per interneuron firing type. For the pyramidal cells, one e-model per layer (2/3, 4, 5, 6) was generated. For e-models that did not generalize well to all the matching morphological types (m-types), additional optimizations were run to generate e-models for specific m-types. This resulted in a final set of 40 e-models.

In the next sections, we present in more details each step of our workflow.

### Electrophysiological features of the SSCx neuronal e-types

**Figure 2:**
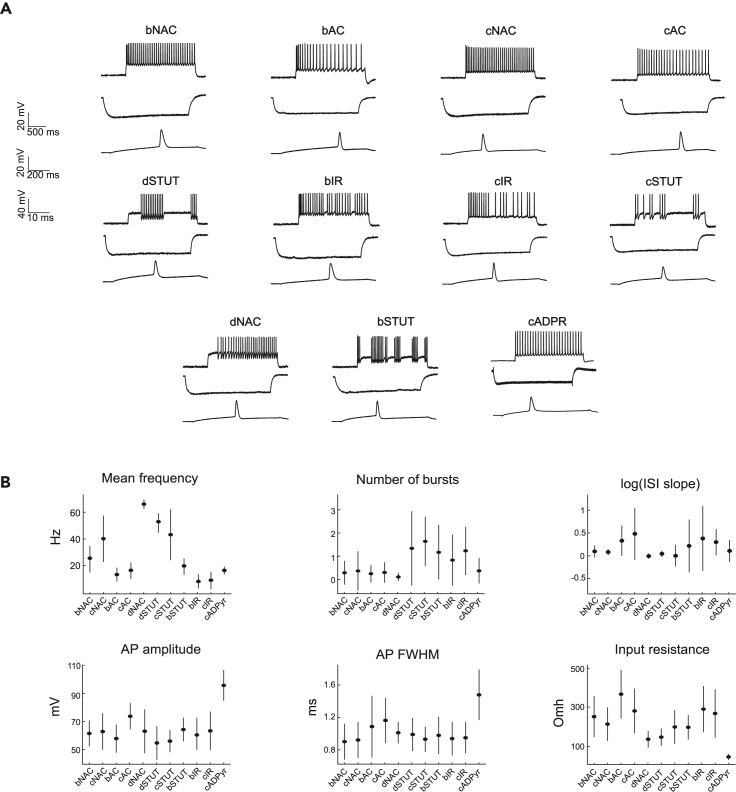
E-features of the SSCx neuronal e-types (A) Exemplar patch-clamp voltage recordings of 11 e-types. Each subplot consists of exemplar traces for three stimuli: long depolarizing current (2 s), hyperpolarizing current (1 s), and a short depolarizing current (50 ms). (B) Exemplar subset of extracted features from experimental recordings for each e-type. The e-features presented here are firing frequency (for injected current corresponding to 300% from the rheobase), number of bursts (current of 150% from the rheobase), logarithm of the slope of ISIs (log(ISI slope); i.e., current of 150% from the rheobase), AP amplitude (current of 150% from the rheobase), AP full width at half maximum (AP FWHM; i.e., current of 150% from the rheobase) and input resistance (current of −40 pA). All features are plotted as mean value ± the standard deviation.

Using the first step of the workflow, we extracted a number of e-features from voltage traces for each neuron that belongs to one of the 11 e-types identified in our SSCx recordings. To demonstrate variability between different e-types, we considered somatic single-cell recordings in response to a depolarizing step current injection (2 s) with different intensities. The features that were extracted from these recordings represent firing properties of the cell, such as the mean firing frequency, interspike intervals (ISIs), and burst number. Then, a hyperpolarizing current (3 s) was applied to characterize passive cell properties and voltage sag. Finally, a short (50 ms) depolarizing step with a high sampling rate was applied to reliably record a single action potential (AP) waveform (Figure 2A). Consequently, features that were extracted from these recordings target single AP properties, such as afterhyperpolarization potential (AHP), AP width, AP fall, and rise time.

To systematically combine e-features from different cells within the same e-type, we deployed the following normalization strategy. First, from the available recordings, we computed a threshold current (rheobase) for each cell. This value was used to re-scale all the protocols (see section “experimental procedures”) such that each protocol corresponds to a percentage of the rheobase. Then, for each chosen protocol intensity (e.g., 150% of the rheobase), we selected the corresponding voltage traces and extracted a set of features from each recording. For each e-type, we then computed the mean and standard deviation of the feature values (Figure 2B), which were used in the next step of the workflow.

We illustrate the diversity of neuronal firing patterns considered in the current study for several extracted e-features in Figure 2B (and Table S1). As expected, pyramidal cells (i.e., cADpyr e-type) exhibit lower input resistance than inhibitory cells. On average, all inhibitory cells have shorter AP duration and lower AP amplitude when compared to cADpyr cells. Among the inhibitory cells, accommodating and irregularly firing cells have lower firing rates than non-accommodating and stuttering ones, while stuttering and irregularly firing types have a higher number of bursts than other firing types. Moreover, typically irregularly firing, bursting, and accommodating e-types show more diversity in their ISIs, reflected in the logarithmic slope of ISIs, than the rest of the firing e-types. This observation is coherent with the firing pattern of the stuttering and irregularly bursting cells, which can display wide variability in the number of bursts and ISIs.

### Model construction and optimization

In order to create an e-model of a neuron, we needed to define ionic mechanisms (see the ion channels and calcium dynamics in Figure 4A), along with previously mentioned experimental constraints and targets (e.g., e-features and reconstructed neuronal morphologies).

The models of ion channels were constructed based on the Hodgkin-Huxley (HH) formalism (see section “experimental procedures” and Figure S1). Each e-model has a number of ionic mechanisms (e.g., persistent and transient sodium, persistent and transient potassium, intracellular calcium dynamics; see Table S3). Several interneuron e-types (bIR, cIR, cSTUT, bSTUT, dSTUT) additionally have a stochastic potassium channel^28^^,^^29^ for irregular spiking. All aforementioned mechanisms were implemented in the NEURON simulator.^30^ A full list of the ionic mechanisms used in this work can be found in Table S5. While models of these ionic mechanisms were defined with respect to their experimental characteristics, some of their parameters, such as the exact maximal ionic conductances or intracellular calcium decay, remain unknown. The aim of the optimization is to find a set of parameters that will allow the model to accurately reproduce cellular features extracted from the experiments.

To solve this task, we used the IBEA algorithm^17^ available in the Python package BluePyOpt^18^ (see section “experimental procedures”). At each generation of the evolutionary process, a wide range of models are produced through random mutations and mating of the members of the previous generations. The score of the offspring is then evaluated by computing the difference between the e-features produced by the e-model and the experimental ones. The parents of the next generation are selected based on their scores. Due to the use of random numbers in the algorithm, this optimization process can be repeated several times with different random seeds until satisfactory models are obtained.

The e-models are evaluated according to a two-step process. First, the resting membrane potential (RMP) and input resistance (Rin) are computed by respectively injecting no stimulus and a small negative stimulus. A binary search algorithm is used to search for the holding current and rheobase by injecting step stimuli with changing amplitudes. Second, the e-models are evaluated for a number of step protocols that reveal the firing properties of the neuron. The current stimuli that are used were individually rescaled based on the holding current and rheobase.

When optimizing interneuron models that contain stochastic potassium channels, we used a two-step optimization. This is due to the fact that the optimization did not converge with all the parameters free at the same time. During the first stage, the stochasticity of the potassium channels was disabled and we optimized for all the e-features except for the burst number. In the second optimization stage, stochasticity was re-enabled and all the parameter values obtained from the first stage were used, except for the maximum conductance of stochastic potassium channels. This part of the optimization used the burst number and ISIs as targets. Such an approach also reduces the optimization time since the non-deterministic mode of stochastic voltage-gated potassium (Kv) channels increases the run time of simulations.

**Figure 3:**
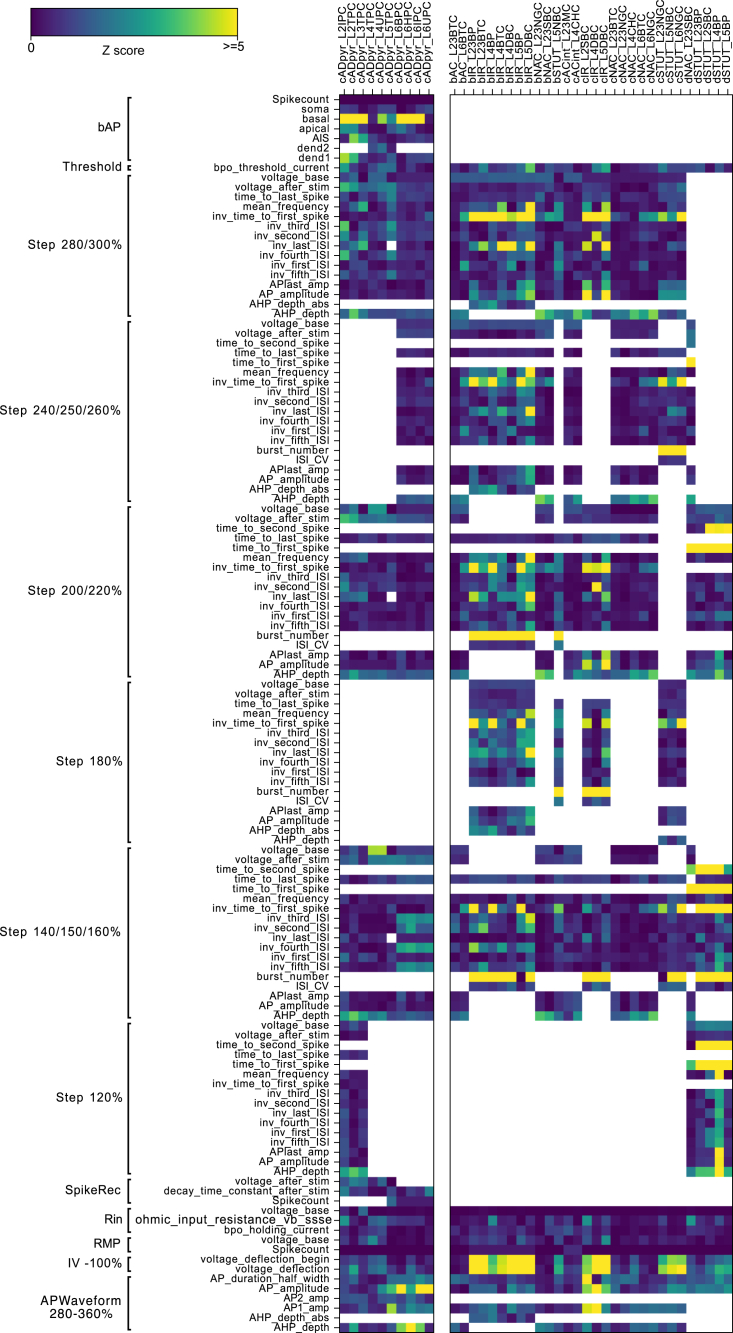
E-feature scores for all e-models optimized E-feature descriptions can be found in Table S6. Blue represents low- and yellow high-cost function values, respectively.

**Figure 4:**
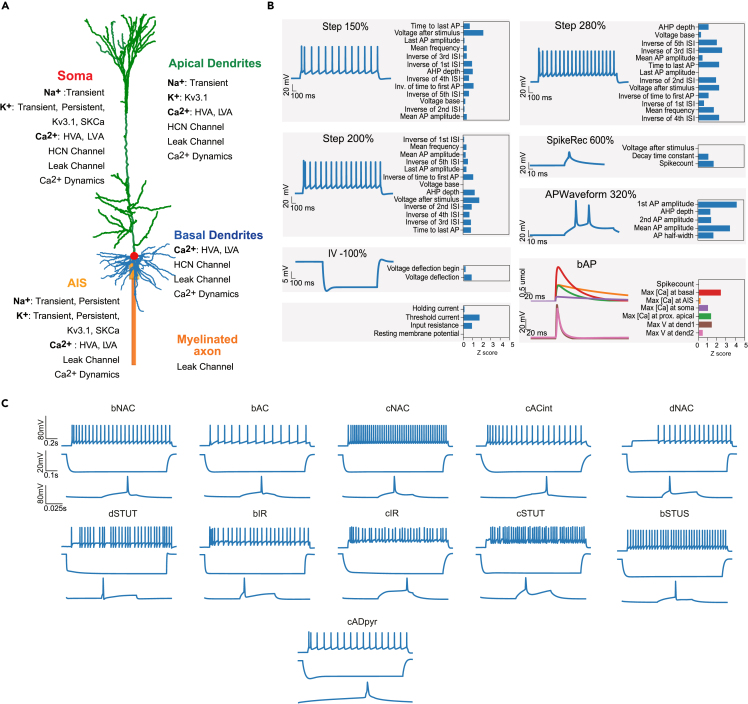
Model construction and optimization results (A) Layer 5 thick-tufted pyramidal cell (L5PC) morphology showing the mechanisms inserted in different morphological sections: the apical dendrites (green), soma (red), basal dendrites (blue), axon initial segment, AIS (thin yellow cylinder), and myelinated axon (thick orange cylinder). (B) Scores and exemplar traces of the optimized L5PC model for e-features of single action potentials (APWaveform), firing properties (Step/IDrest), input resistance and hyperpolarization features (IV), back-propagating action potential and peak intracellular calcium concentration recorded in the apical dendrites, soma and AIS, (bAP, for pyramidal neurons only), and spike recovery (SpikeRec). The resting membrane potential (RMP), holding current, and threshold current are also optimized as e-features. (C) *In silico* voltage recordings obtained from e-models (one for each e-type) for three protocols: IDrest (150%/140% from the rheobase), IV (−100% from the rheobase), and APWaveform (320%/350% from the rheobase). These e-models are in close agreement with various experimental e-types, as shown in Figure 2A.

Using this procedure, we optimized e-models for all aforementioned SSCx e-types with a specific set of e-features for each e-type (optimization scores are reported in Figure 3, for the list of e-features and protocols see Table 1). Exemplar responses from different e-models representing all the e-types are depicted in Figure 4C. Note the similarity in the firing patterns between the e-models and the experimentally recorded data (Figure 2A). The results of the optimizations and corresponding voltage traces are illustrated for a thick-tufted pyramidal neuron e-model (cADpyr L5TPC) in Figure 4B. The majority of e-feature scores (*Z* scores) for this e-model are below two standard deviations of the experimental mean value, suggesting the model closely replicates the experimental data. The e-model was optimized for multiple starting seed values and the best seed was chosen for each e-model. Once the best e-model is found for an e-type, it undergoes validation.

**Table 1:** List of protocol and features used for optimizations for each e-type

E-type	Protocol	E-features
Pyramidal cells	APWaveform 320%	AP _ amplitude, AP1 _ amp, AP2 _ amp, AP _ duration _ half _ width, AHP _ depth
	IV -100%	voltage _ deflection, voltage _ deflection _ begin
	IDrest and IDthresh 150%, 200%, 280%	voltage _ base, voltage _ after _ stim, AP _ amplitude, APlast _ amp, AHP _ depth, inv _ time _ to _ first _ spike, time _ to _ last _ spike, inv _ first _ ISI, inv _ second _ ISI, inv _ third _ ISI, inv _ fourth _ ISI, inv _ fifth _ ISI, mean _ frequency
	SpikeRec 600%	decay _ time _ constant _ after _ stim, voltage _ after _ stim, Spikecount
	IV −20% (Rin)	ohmic _ input _ resistance _ vb _ ssse, voltage _ base
	IV 0% (RMP)	voltage _ base, Spikecount
	RinHoldCurrent	bpo _ holding _ current
	Threshold	bpo _ threshold _ current
	bAP	Spikecount, maximum _ voltage _ from _ voltagebase, maximum _ ca _ prox _ apic _ from _ voltagebase, maximum _ ca _ prox _ basal _ from _ voltagebase, maximum _ ca _ prox _ soma _ from _ voltagebase, maximum _ ca _ prox _ ais _ from _ voltagebase
bAC, cAC	IDThresh/IDrest 140%, 200%, 250%, 300%	voltage _ base, voltage _ after _ stim, AP _ amplitude, APlast _ amp, AHP _ depth,inv _ time _ to _ first _ spike, time _ to _ last _ spike, inv _ first _ ISI, inv _ second _ ISI, inv _ third _ ISI, inv _ fourth _ ISI, inv _ fifth _ ISI, mean _ frequency
	APWaveform 360%	AP _ amplitude, AP1 _ amp, AP _ duration _ half _ width, AHP _ depth
	IV −100%	voltage _ deflection, voltage _ deflection _ begin
	IV −20% (Rin)	ohmic _ input _ resistance _ vb _ ssse, voltage _ base
	IV 0% (RMP)	voltage _ base, Spikecount
	RinHoldCurrent	bpo _ holding _ current
	threshold	bpo _ threshold _ current
bNAC, cNAC	IDThresh/IDrest 150%, 200%, 250%, 300%	voltage _ base, voltage _ after _ stim, AP _ amplitude, APlast _ amp, AHP _ depth, inv _ time _ to _ first _ spike, time _ to _ last _ spike, inv _ first _ ISI, inv _ second _ ISI, inv _ third _ ISI, inv _ fourth _ ISI, inv _ fifth _ ISI, mean _ frequency
	APWaveform 360%	AP _ amplitude, AP1 _ amp, AP _ duration _ half _ width, AHP _ depth
	IV −100%	voltage _ deflection, voltage _ deflection _ begin
	IV −20% (Rin)	ohmic _ input _ resistance _ vb _ ssse, voltage _ base
	IV 0% (RMP)	voltage _ base, Spikecount
	RinHoldCurrent	bpo _ holding _ current
	threshold	bpo _ threshold _ current
dNAC	IDThresh/IDrest 120%, 150%, 200%, 260%	voltage _ base, voltage _ after _ stim, AP _ amplitude, APlast _ amp, AHP _ depth, inv _ time _ to _ first _ spike, time _ to _ last _ spike, inv _ first _ ISI, inv _ second _ ISI, inv _ third _ ISI, inv _ fourth _ ISI, inv _ fifth _ ISI, mean _ frequency
	APWaveform 300%	AP _ amplitude, AP _ duration _ half _ width
	IV −100%	voltage _ deflection, voltage _ deflection _ begin
	IV −20% (Rin)	ohmic _ input _ resistance _ vb _ ssse, voltage _ base
	IV 0% (RMP)	voltage _ base,Spikecount
	RinHoldCurrent	bpo _ holding _ current
	threshold	bpo _ threshold _ current
cSTUT	IDThresh/IDrest 180%, 280%	voltage _ base, voltage _ after _ stim, AP _ amplitude, APlast _ amp, AHP _ depth, inv _ time _ to _ first _ spike, time _ to _ last _ spike, inv _ first _ ISI, inv _ second _ ISI, inv _ third _ ISI, inv _ fourth _ ISI, inv _ fifth _ ISI, inv _ last _ ISI, mean _ frequency
	IDThresh/IDrest 140%, 240%	mean _ frequency, inv _ time _ to _ first _ spike, time _ to _ last _ spike, inv _ first _ ISI, inv _ second _ ISI, inv _ third _ ISI, inv _ fourth _ ISI, inv _ fifth _ ISI, inv _ last _ ISI, burst _ number, ISI _ CV
	APWaveform 320%	AP _ amplitude, AP1 _ amp, AP _ duration _ half _ width, AHP _ depth
	IV −100%	voltage _ deflection, voltage _ deflection _ begin
	IV −20% (Rin)	ohmic _ input _ resistance _ vb _ ssse, voltage _ base
	IV 0% (RMP)	voltage _ base, Spikecount
	RinHoldCurrent	bpo _ holding _ current
	threshold	bpo _ threshold _ current
bSTUT	IDThresh/IDrest 140%, 280%	voltage _ base, voltage _ after _ stim, AP _ amplitude, APlast _ amp, AHP _ depth, inv _ time _ to _ first _ spike, time _ to _ last _ spike, inv _ first _ ISI, inv _ second _ ISI, inv _ third _ ISI, inv _ fourth _ ISI, inv _ fifth _ ISI, inv _ last _ ISI, mean _ frequency
	IDThresh/IDrest 180%, 220%	mean _ frequency, inv _ time _ to _ first _ spike, time _ to _ last _ spike, inv _ first _ ISI, inv _ second _ ISI, inv _ third _ ISI, inv _ fourth _ ISI, inv _ fifth _ ISI, inv _ last _ ISI, burst _ number, ISI _ CV
	APWaveform 300%	AP _ amplitude, AP1 _ amp, AP _ duration _ half _ width, AHP _ depth
	IV −100%	voltage _ deflection, voltage _ deflection _ begin
	IV −20% (Rin)	ohmic _ input _ resistance _ vb _ ssse, voltage _ base
	IV 0% (RMP)	voltage _ base,Spikecount
	RinHoldCurrent	bpo _ holding _ current
	threshold	bpo _ threshold _ current
dSTUT	IDThresh/IDrest 120%, 200%	voltage _ base, voltage _ after _ stim, AP _ amplitude, APlast _ amp, AHP _ depth, inv _ time _ to _ first _ spike, time _ to _ last _ spike, inv _ first _ ISI, inv _ second _ ISI, inv _ third _ ISI, inv _ fourth _ ISI, inv _ fifth _ ISI, inv _ last _ ISI, mean _ frequency
	IDThresh/IDrest 140%	mean _ frequency, inv _ time _ to _ first _ spike, time _ to _ last _ spike, inv _ first _ ISI, inv _ second _ ISI, inv _ third _ ISI, inv _ fourth _ ISI, inv _ fifth _ ISI, inv _ last _ ISI, burst _ number, ISI _ CV
	APWaveform 300%	AP _ amplitude, AP1 _ amp,
		AP _ duration _ half _ width, AHP _ depth
	IV −100%	voltage _ deflection, voltage _ deflection _ begin
	IV −20% (Rin)	ohmic _ input _ resistance _ vb _ ssse, voltage _ base
	IV 0% (RMP)	voltage _ base,Spikecount
	RinHoldCurrent	bpo _ holding _ current
	threshold	bpo _ threshold _ current
cIR	IDThresh/IDrest 220%, 280%	voltage _ base, voltage _ after _ stim, AP _ amplitude, APlast _ amp, AHP _ depth, inv _ time _ to _ first _ spike, time _ to _ last _ spike, inv _ first _ ISI, inv _ second _ ISI, inv _ third _ ISI, inv _ fourth _ ISI, inv _ fifth _ ISI, inv _ last _ ISI, mean _ frequency
	IDThresh/IDrest 140%, 180%	mean _ frequency,inv _ time _ to _ first _ spike, time _ to _ last _ spike, inv _ first _ ISI, inv _ second _ ISI, inv _ third _ ISI, inv _ fourth _ ISI, inv _ fifth _ ISI, inv _ last _ ISI, burst _ number, ISI _ CV
	APWaveform 280%	AP _ amplitude, AP1 _ amp, AP _ duration _ half _ width, AHP _ depth
	IV −100%	voltage _ deflection, voltage _ deflection _ begin
	IV −20% (Rin)	ohmic _ input _ resistance _ vb _ ssse, voltage _ base
	IV 0% (RMP)	voltage _ base,Spikecount
	RinHoldCurrent	bpo _ holding _ current
	threshold	bpo _ threshold _ current
bIR	IDThresh/IDrest 180%, 240%, 280%	voltage _ base, voltage _ after _ stim, AP _ amplitude, APlast _ amp, inv _ time _ to _ first _ spike, time _ to _ last _ spike, inv _ first _ ISI, inv _ second _ ISI, inv _ third _ ISI, inv _ fourth _ ISI, inv _ fifth _ ISI, inv _ last _ ISI, AHP _ depth _ abs, mean _ frequency
	IDThresh/IDrest 140%, 200%	mean _ frequency, inv _ time _ to _ first _ spike, time _ to _ last _ spike, inv _ first _ ISI, inv _ second _ ISI, inv _ third _ ISI, inv _ fourth _ ISI, inv _ fifth _ ISI, inv _ last _ ISI, burst _ number, ISI _ CV
	APWaveform 280%	AP _ amplitude, AP1 _ amp, AP _ duration _ half _ width, AHP _ depth _ abs
	IV −100%	voltage _ deflection, voltage _ deflection _ begin
	IV −20% (Rin)	ohmic _ input _ resistance _ vb _ ssse, voltage _ base
	IV 0% (RMP)	voltage _ base,Spikecount
	RinHoldCurrent	bpo _ holding _ current
	threshold	bpo _ threshold _ current

### Validation and analysis of the detailed neuronal model

It was unknown how the optimized e-models would respond to the stimuli not used in the process of optimization. To test this, we applied two validation routines to the study case of L5PC: dendro-synaptic^11^ and somatic. Since dendritic attenuation data were available only for the L5PC, the validation and e-model analyses were performed only for this e-model.

**Figure 5:**
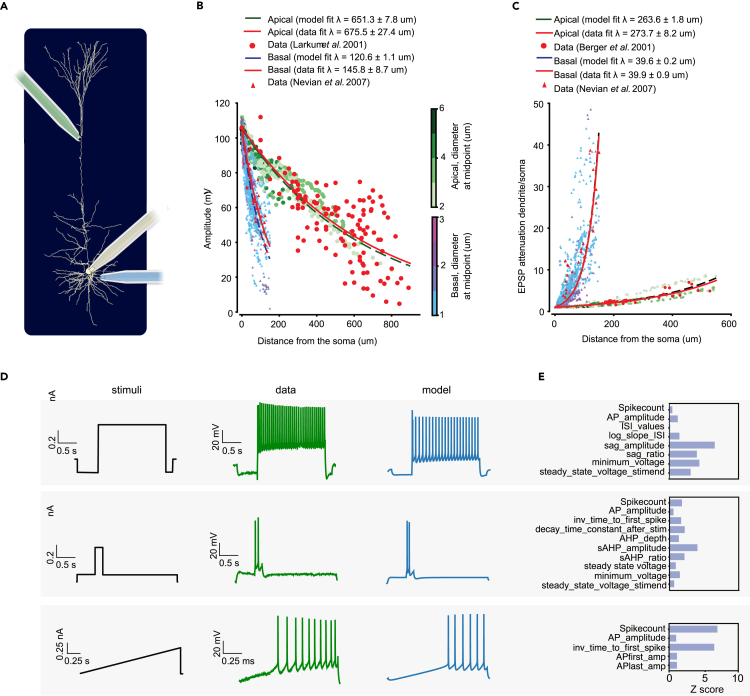
Synaptic and somatic validations of L5TPC electrical model (A) Schematic representation of the experimental setup. (B) Predicted bAP amplitude measured at different locations on apical (green dots) or basal (blue dots) dendrites and experimental results from literature (red dots). The data were fitted with an exponential for the *in silico* (black dashed lines) and experimental (red dashed lines) results. The color bar indicates the diameter of apical and basal dendrites at different distances from soma. (C) Predicted dendritic to somatic attenuation ratios for *in silico* EPSP amplitudes measured at different locations on apical (green dots) and basal (blue dots) dendrites. Color code is similar to (B). (D) Somatic validation. For each injected somatic stimulus (the left-most column, black) an exemplar voltage trace is plotted from the recorded data (middle column, green) and from the e-model’s response (the right-most column, blue). (E) Feature scores calculated based on the model responses and experimental recordings in (D).

First, we tested whether our model can reproduce the attenuation of dendritic back-propagating APs (bAPs) and excitatory postsynaptic potentials (EPSPs) observed in the literature. For bAP attenuation, we injected a current step (5 ms, 2 nA) in the soma and recorded the voltage from apical and basal dendrites at various distances from the soma (Figure 5A). We compared the attenuation length constant of our *in silico* results to the experimentally reported ones^31^^,^^32^ (Figure 5B). For validation of EPSP attenuation, we simulated a transient change in the synaptic conductance in distant apical (1.5 nS) and basal (0.2 nS) dendrites (Figure 5C). Then, we calculated the ratio of dendritic to somatic attenuation and compared it to the experimental results reported previously.^31^^,^^32^ Both bAP and EPSP attenuation in the basal dendrites in the e-model (bAP, 120.6±1.1 μm; EPSP, 39.6±0.2 μm) were consistent with the experimentally reported values (bAP, 145.8±8.7 μm; EPSP, 39.9±0.9 μm). In the case of the apical dendrite, agreement between *in silico* (bAP, 651.3±1.8; EPSP, 263.6±1.8 μm) and experimental (bAP, 675.5.3±27.4 μm; EPSP, 273.7±8.2 μm) results were achieved for the *in silico* morphologies with apical dendrites with a diameter of 2−6 μm (Figures 5B and 5C). These diameter values are larger than those found in the juvenile rat,^33^ which is consistent with the fact that the aforementioned studies were performed in the adult rat. The *in silico* standard deviations of the dendritic validations are about 20 times lower than the *ex vivo* ones, possibly due to noise in the experimental data or variability in dendrite diameter. This discrepancy might also stem from using a fixed electrical model *in silico*, compared to the unique neuronal conductances in each *ex vivo* cell.

Next, to test whether the e-model could reproduce a wide range of somatic responses, we used somatic validations. We aimed to compare the experimental data to the responses of the optimized model for stimuli that were not used in the course of optimizations.^34^ We considered three protocols (Ramp, sAHP, and IDHyperpol; see Table 2 section “experimental procedures”) (Figure 5D, first column). From the corresponding single-cell recordings (Figure 5D, second column) and model responses (Figure 5D, third column) we extracted a set of e-features and calculated their scores (Figure 5E). Most of the e-features of the e-model are less than five standard deviations away from the experimental e-features, except the “spike count” and “time to first spike” for the Ramp protocol (eight standard deviations instead).

**Table 2:** List of protocols and e-features used for validations of the L5PC model

Protocol	E-features
IDHyperpol 250%	Spikecount, AP _ amplitude, ISI _ values, ISI _ log _ slope, sag _ amplitude, sag _ ratio, minimum _ voltage, steady _ state _ voltage _ stimend
sAHP 150, 350%	Spikecount, AP _ amplitude, inv _ time _ to _ first _ spike, AHP _ depth _ abs, sag _ ratio, decay _ time _ constant _ after _ stim, steady _ state _ voltage _ stimend, sag _ ratio, minimum _ voltage, steady _ state _ voltage
APThresh 150%	Spikecount, AP _ amplitude, inv _ first _ ISI, AP1 _ amp, APlast _ amp

**Figure 6:**
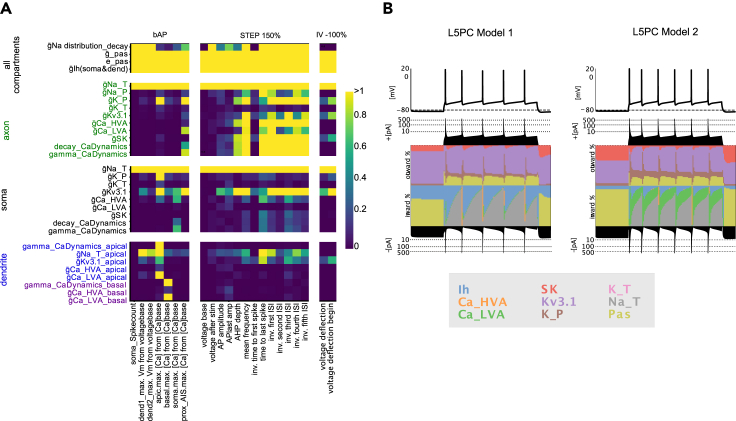
Sensitivity analysis and degeneracy (A) Analysis of the sensitivity of e-features to changes in the parameter values. The matrix represents slopes of e-features values. The sensitivity is presented for e-features extracted based on three protocols (bAP, step 150% and IV −100%). Colors reflect the value of the slope, with slopes greater than 1 represented in yellow. (B) Currentscape plots of two L5PC e-models, with two different sets of maximal intrinsic conductances. Top: model responses to the same current stimuli (step 150%). Bottom: black-filled plots represent total positive (top) and negative (bottom) currents in the cell during the stimuli. The middle panel represents the contribution of ionic inward and outward currents during the stimuli, and each color curve reveals the contribution of one particular ionic current as the percentage of the total current during the simulation.

To assess whether the parameters of the L5PC e-model were well restricted by the e-features used in optimization, we analyzed the parameter sensitivity of the e-model (Figure 6A). For this, we varied the value of one parameter at a time while keeping the rest of the parameters fixed. The values of the parameters were varied by 1%, 50%, and 90%. Then, we computed the slope of the change in score value for each parameter (Figure S2). If the slope was approximately zero, it meant that changes in the parameter value did not affect the e-feature in question. We demonstrate the results of this analysis for three protocols (bAP, Step_150%, and IV_-100%), to reveal involvement of neuronal model parameters in dendritic, somatic firing, and hyperpolarizing neuronal response. The same type of analysis can be applied to the different protocols and e-features to study more intricate details of the parameter importance. Our results show that the passive parameters ($\bar{g}$_pas, e_pas, $\bar{g}$ Ih), axonal sodium and somatic sodium demonstrated a slope larger than 1 for all e-features in all the protocols considered (Figure 6A). All axonal parameters except transient potassium, somatic sodium, and $\bar{g}$ Kv1.3 also show a slope larger than 1 for the e-features reflecting firing properties of the cell, such as ISIs and firing frequency (Figure 6A). The level of calcium at the apical point was affected by manipulation of maximum conductances of apical sodium, low threshold calcium, and calcium dynamics, while less by high threshold calcium channels (slope = 0.16). All e-features show low sensitivity to the following parameters: somatic and axonal $\bar{g}$ K_T (maximum slopes are 0.2 for the apical calcium amplitude and 0.16 for the time to last spike feature), somatic $\bar{g}$ Ca_LVA (slope, 0.14 for last AP amplitude), and apical $\bar{g}$ Ca_HVA (slope, 0.16 for feature amplitude of calcium in apical dendrite; Figure 6A). These results indicate that the model is sensitive to changes in the majority of the parameters (26 out of 31).

Finally, we tested whether our modeling approach can reproduce the phenomenon of the degeneracy of the ionic currents, which was previously reported to take place in electrical neuron models.^35^^,^^36^^,^^37^^,^^38^^,^^39^^,^^40^ For this purpose, we optimized another L5PC e-model, using different seeds for initialization of the e-model parameters. To illustrate the difference between the e-models, we created currentscape plots^41^ of the somatic currents in response to the same percentage of injected current (depolarizing step of 150% rheobase) (Figure 6B). We observed that the second e-model has a more prominent contribution of the Ca_LVA and less contribution of sodium current during firing than the first e-model. The difference between the e-models was also present in the contribution of potassium currents (K_P, Kv1.3, SK) and Ih. However, both e-models did not have any contribution of transient potassium (K_T) to the resting or firing states. This might indicate that K_T is not well restricted by the features used for optimization, independent of the optimization seed.

### Generalization of electrical models

Each of the aforementioned optimized e-models was built for a particular morphology, which we call the exemplar morphology. When these e-models are meant to be used in a large network model, the question of the generalization of an e-model to other morphologies of the same or of different morphological type arises. Given the computational cost of optimizing an e-model and the possibly large number of reconstructed morphologies in the network, we chose to take the following approach. We assigned to each morphology the e-models that match it best, based on the most common e-types for a certain m-type.

An e-model for a morphology is accepted according to the rule in Equation 2, following the approach of Markram et al.^11^ For this purpose, we used a total of 1,015 reconstructed and manually corrected morphologies of SSCx young rat cortex for the generalization routine (see section “experimental procedures”). The population of morphologies was artificially increased to 141,733 by a cloning procedure on the reconstructions.^42^ Given their m-type, morphologies were then attributed to one of the possible morpho-electro combinations (me-combinations)^11^^,^^24^ (see section “experimental procedures”), yielding 366,926 models, of which 233,941 passed generalization.

**Figure 7:**
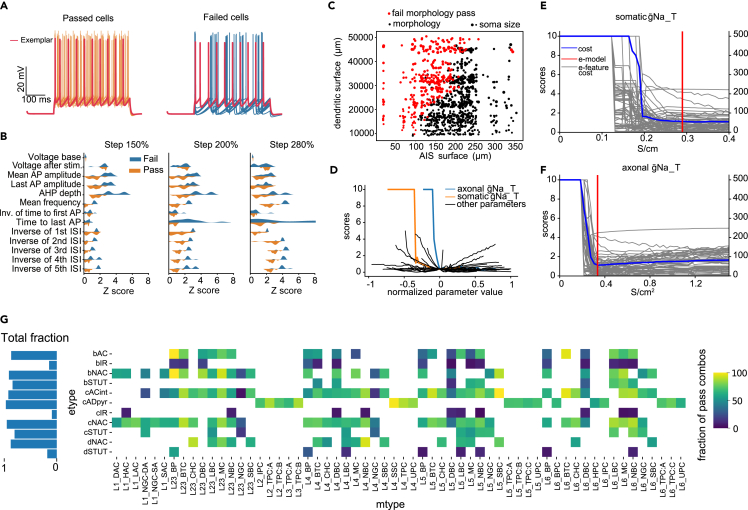
Generalization of electrical models (A) Examples of the traces for L5PC me-combination, in response to the depolarizing step of 150%. Traces of me-combinations that pass (left, orange and red traces) and (right, blue and red traces) the generalization procedure. Red traces correspond to the optimized canonical e-model. (B) Example of e-feature scores for me-combos that pass (orange) and fail (blue) generalization. E-features were extracted for three depolarization protocols (steps 150%, 200%, 280%). (C) Example of the morphological properties of L5PC me-combinations that passed and failed generalization. Plot illustrates the relation between the total surface area of the AIS (axon up to 40μm) and the total surface area of the proximal dendritic compartments (up to 500μm in path length) for 1,000 randomly sampled L5PC morphologies. Red dots represent failed morphologies, black dots represent passed morphologies. Size of dots represents size of the soma. (D) Parameter sensitivity analysis for the firing frequency e-feature in response to the depolarizing stimuli (150%). Dependency of e-feature score is plotted versus normalized value of the parameters. The blue line represents axonal sodium, orange represents somatic sodium, black lines represent all other parameters. Maximum score is clipped at 10. (E and F) Sensitivity analysis with the exemplar morphology (similar to Figure 6) of the sodium conductance parameter in the axon (F) and soma (E) on the cost (red) and all features (gray). Red represents the value of these parameters for this cADpyr e-model (0.33 for axon and 0.29 for soma). (G) For each combination of m-type and e-type, we display the fraction of accepted cells, with total fraction for each e-type on the left.

For the L5PC e-model, we ran a generalization routine and separated morphologies on those that passed and failed this procedure (Figure 7A). We compared the distributions of e-feature scores between populations of morphologies that failed and passed generalization (Figure 7B). We show that the e-features responsible for rejection of the morphologies are mostly related to firing properties and after-hyperpolarization depth.

**Figure 8:**
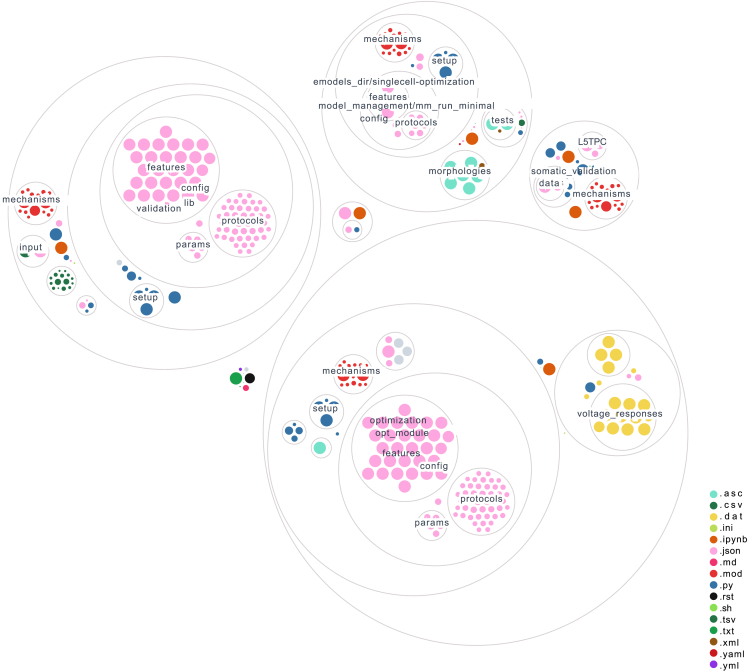
A visual representation of the code base Orange circles indicate the interactive Python notebooks, while blue circles represent the Python modules. Pink circles correspond to the files that contain parameters for all cell types in the SSCx. Although the full demonstration primarily focuses on the L5PC example, we still provide the configuration for all other cell types as a resource for the community.

To gain a deeper insight into why some morphologies were not accepted for the L5PC e-model, we had a closer look at two morphological features (Figure 7C), the surface area of the axon initial segment (AIS)—taken here as the first 40μm of axon—versus the proximal dendritic surface area (up to 500μm in path length) for a random sample of 1,000 morphologies. As the APs are generated in the AIS before triggering ion channels in the soma, the surface areas of these two compartments are expected to be the most important for the firing properties of a cell. Indeed, we observe a near-linear correlation between these two quantities for a morphology to be valid under this e-model. These results are in line with previous works,^43^^,^^44^ which have shown that a consistent ρ factor, defined as the ratio of input resistances between the AIS and somatodendritic compartments, is important to ensure generalizability of electrical models. Interestingly we noticed that soma surface area does not correlate with the pass/fail of the cell (Figure 7C).

Further, we confirm that the sodium channels ($\bar{g}$ Na_T somatic and axonal) are indeed the most important for firing e-features such as spiking frequency (Figure 7D). These two sodium channels are able to bring the cell to a regime where rheobase is too high with respect to experimental recordings, and thus no protocols can even be computed, resulting in maximum scores. For these two conductance parameters, we show in Figure 7E (for soma) and Figure 7F (for axon) the sensitivity to all other e-features as well as the total cost (used for optimization) as a function of the non-normalized conductance values. We observe that the optimal solution is near the lowest possible $\bar{g}$ Na_T for the exemplar cell to fire, and thus any small deviation of the ratio between the size of the axon and the rest of the morphology may bring a cell into a non-firing regime. This transition point corresponds to the diagonal separation between black and red dots in Figure 7C, as already pointed out in the work by Hay and collegues.^43^ On the contrary, the $\bar{g}$ Na_T in the soma in Figure 7E is further away from the non-firing regime, and thus the soma size is less important for a cell to be generalized on other morphologies. The optimized value of the axonal $\bar{g}$ Na_T is close to the minimal value for the exemplar cell to be valid. Thus, if a morphology with the same dendritic structure has an AIS with a smaller surface area, the cell will not pass our selection criteria.

With this morphological and parameter sensitivity analysis, we were able to determine that the low value of axonal $\bar{g}$ Na_T was the main cause of failed morphologies, suggesting that a slight manual adjustment of this parameter may help to improve the generalizability of this e-model.

Overall, our pipeline produces generalizable electrical models with a high acceptance rate (cADpyr, 73%; cNAC, 71%; cACint, 69%; bNAC, 68%; dNAC, 66%; bAC, 65%; bSTUT, 63%; cSTUT, 63%) for most e-types, except for three irregular electrical types (dSTUT, 13%; bIR, 10%; cIR, 6%): bIR, cIR, and dSTUT (Figure 7G).

For the dSTUT e-type, only a few me-models showed a delay in the first spikes (not shown), preventing most from passing. For cIR and bIR e-types, the spiking frequencies and ISIs are the most frequent e-features to fail, in addition to the burst number for bIR (meaning many me-models did not burst as expected) and the amplitudes of APs for cIR (not shown).

Finally, as a means to verify that the e-models obtained in this work are more generalizable than in a previous study,^11^ we compared the results of generalizability between both versions and showed a clear improvement using the present e-models (see supplemental information and Figure S3).

## Discussion

In this work, we present an automatic workflow for single-cell model creation. We demonstrate the application of this workflow by producing 40 e-models for 11 e-types of the somatosensory cortex of a juvenile rat. These models reproduce neuronal responses from patch-clamp recordings observed in the corresponding cells. As an example of a single-cell study, we assessed the L5PC e-model robustness, analyzed its parameters, and tested the morphological generalization. We show that the optimized L5PC e-model can be successfully generalized to a wide range of L5PC morphologies and that the majority of the e-models parameters are constrained by the optimization cost function used in this study. Moreover, we show that L5PC models can reliably reproduce dendritic signal attenuation. These canonical e-models can be used to study signal propagation in a single cell or in networks. All the steps of the workflow are available to the reader as openly accessible Python notebooks (see section “data and code availability”).

The approach of building a canonical e-model is based on averaging neuronal responses across different cells that belong to the same e-type. Although we observe certain variability in the extracted e-features for each e-type (Figure 2), the canonical e-models will produce only a single e-feature value. This implies that the variability present in the data is dismissed. This is not necessarily prohibitive in studies and analyses of average neuronal behavior but can be problematic in tasks where variability is implied, such as circuit simulations. One direct way to overcome this limitation is to build e-models for every single neuron in the network. However, this would require a large amount of experimental data and be computationally expensive. It would also be possible to generate several e-models based on the same input, such that the resulting e-models have different optimized sets of parameters and therefore could represent the variability present in the data. In the current work, we use the IBEA algorithm to optimize neuronal parameters; this algorithm employs a stochastic process that randomly generates candidate solutions to a given optimization problem. Therefore, using different random seeds can lead to different solutions in the IBEA algorithm since they cause the algorithm to explore different regions of the solution space, which can result in varied outcomes. Launching optimizations with several different random seeds will allow for the creation of several canonical e-models, each from the same population, yet allowing for the variability of neuronal responses. Similarly, one could leverage Bayesian statistics tools to generate a large number of the single-neuron parameters.^45^ Alternatively, we introduce variability among the me-models by generalizing the e-models over a large set of morphologies. We have shown that, by applying different morphologies to the canonical e-models, we introduced variability in the resulting e-feature values (Figure 7B). This is possible because the conductances were optimized for one morphology and certain parameters are sensitive to the change of the morphological characteristics of the cell (e.g., Ih current to the size of the dendrite).

The e-models described in this paper can be used for a somatosensory cortex network model. We aim to adapt and apply our automatic workflow for single-cell model creation in upcoming network models of other brain regions. To ensure compatibility and seamless integration, we are adopting appropriate standards and methodologies tailored to the specific requirements of these models and brain regions. By doing so, we anticipate a broader applicability of our workflow, enabling researchers to study neuronal responses across a diverse range of brain areas. Furthermore, the open accessibility of our workflow through Python notebooks encourages collaboration and continuous improvement within the scientific community, allowing for more refined and accurate models to be developed in the future.

The set of e-models produced in this work is based on somatic patch-clamp recordings and can be enriched with more biophysical details in future versions. For example, to allow for a study of neuronal synaptic and dendritic integration, the current models of L5PC would benefit from constraining the e-model by using dendritic calcium imaging data. Also, several currents and channels could be included in the models, such as specific small- and large-conductance dendritic channels,^46^^,^^47^ or a nonlinear A-type K+ current distribution along the dendrites.^48^^,^^49^ Moreover, by including axonal reconstructions and fitting corresponding axonal conductances, we could better understand their role in spike propagation and cell signaling.

In the present case of the SSCx interneuron optimizations, we were able to successfully reproduce somatic responses of a wide range of e-types. For the irregular and stuttering interneurons, this was only possible by including stochastic potassium channels in the models. Interneuron e-models could be further refined through somatic validation analysis by quantifying the performance of optimized e-models on a battery of recordings that were not used in optimizations. This could be achieved using various stimuli, such as a ramp or a sinusoid. Finally, it should be mentioned that another approach to produce even more detailed e-models would be to incorporate transcriptomics data,^22^^,^^25^^,^^50^ which would guide the composition of ionic currents present in the model. However, it is not clear yet how much the gene-level expression is an indicator of the protein densities on the cellular membrane.

Yet another aspect of our approach to building a canonical e-model was that the optimization was performed on a single morphology, while the variability of morphologies is present within each m-type.^51^^,^^52^^,^^53^ We explored how well a single canonical e-model can be generalized on various morphologies of compatible m-types. According to our results, while other interneurons had high total generalizability, irregularly firing and delayed-stuttering interneurons showed poor generalizability. This could indicate that irregularly firing models are more sensitive than, for example, continuously firing e-types, to the morphological properties of the cells. We also observed that pyramidal cells show high generalizability. When we analyzed the morphological properties that allowed L5PC e-models to perform acceptably on a large set of morphologies, we noticed that the size of the AIS and the dendritic area play a considerable role in the e-model score. Most of the morphologies with a small AIS and a large dendritic area failed when applied to the canonical e-model, which is consistent with previously reported results.^43^ To further understand and possibly improve the generalizability of canonical e-models, one would need to study the link between morphological properties and electrical features of the e-models in more depth.^54^

The parameters of the optimized e-model can be analyzed to assess its biophysical properties. For example, with sensitivity analysis, we showed how the performance of the models is affected by the perturbations of the parameters.^55^^,^^56^ In the case of the L5PC e-model, most of the parameters were constrained by the chosen evaluation function, while changes in some parameters, such as axonal and somatic persistent potassium current maximal conductances, had no effect on the e-features. This type of analysis may guide choices for e-features and protocols to consider in the score function, such that all parameters are sufficiently constrained. For a more in-depth analysis, a closer look at the channel kinetics and its interplay with the rest of the currents in the cell may provide us with more information about e-model sensitivity. Another aspect of the parameter space analysis is to look at the degeneracy of parameters,^35^^,^^36^^,^^40^ meaning that we can produce several parameter sets that would result in similar performances of the e-model. We used currentscapes plots to visualize the current dynamics of two L5PC models with similar performance (reflected in the values of the evaluation function). We saw that ionic contributions to the overall current were different between the models. However, both models have a similar parameter sensitivity, with, for example, no contribution of persistent potassium current. Further, it could be interesting to investigate to what extent the definition of the score function can affect the presence of degeneracy in the system and to what extent it is an implicit property of the system. Overall, this approach can be used to shine a light on several aspects of neuronal physiology. Sensitivity analysis can be used to probe the contribution of each ion current to the e-feature of interest. This allows us to understand cellular behavior in various conditions and potentially manipulate it. For example, one could examine the role of ion channels in change of intrinsic neuronal excitability, a common feature in conditions such as epilepsy,^57^^,^^58^ schizophrenia,^59^ and Alzheimer’s disease.^60^^,^^61^ Identifying channels that specifically influence hyperexcitability can lead to new hypotheses generation and testing. Finally, understanding biophysical principles behind neuronal heterogeneity can help us to get more insight into neuronal resilience to perturbations.

### Limitations of the study

In the present study, we preset an automated workflow for single-cell model development and its applications, and, while promising, it is not devoid of limitations. First of all, our approach to optimization is based on usage of a single morphology, neglecting the inherent morphological variation in each m-type. This was demonstrated in our generalizability tests across various morphologies of compatible m-types, with specific interneurons manifesting poor generalizability. Furthermore, the foundation of our e-models lies in somatic patch-clamp recordings. Future iterations of these models are necessary to gain a more biophysically detailed perspective. A salient limitation, akin to detailed biophysical cell modeling endeavors, is our reliance on the phenomenological HH models for ionic currents within the cell. This overlooks a more granular and precise representation of cellular biological processes.

## Experimental procedures

### Resource availability

#### Lead contact

Further information and requests for resources should be directed to and will be fulfilled by the lead contact, Werner Van Geit (werner.vangeit@gmail.com).

#### Materials availability

This study did not generate any new reagents or materials.

### Data: Electrophysiological recordings and morphologies

#### Electrophysiological recordings

Electrophysiological recordings were obtained from P14–16 rat somatosensory cortex using whole-cell patch-clamp experiments. The recordings were performed as described in Markram et al.^11^ Each cell was classified according to its firing type based on the Petilla convention.^11^^,^^23^ Recordings were performed in pyramidal cells (seven L6PCs, 44 L5PCs, three L4PCs, eight L23PCs) and interneurons (16 cACs, 22 bACs, 19 cNACs, 28 bNACs, seven dNACs, 11 dSTUTs, eight bSTUTs, 10 cSTUT, 14 bIR, six cIRs). A number of stimuli were applied for each cell: IDrest (depolarizing steps, sampling frequency, 10 kHz; duration, 2s), IDthresh (depolarizing steps, sampling frequency, 10 kHz; duration, 2 s), APWaveform (depolarizing steps, sampling frequency, 50 kHz; duration, 50 ms), IV (sequence of current steps, from hyperpolarization to depolarization, sampling frequency, 10 kHz; duration, 3 s), SpikeRec (sequence of brief depolarizing pairs with increasing interval, sampling frequency, 50 kHz; duration, 1.5 s), Ramp (ramp current, sampling frequency, 10 kHz; duration, 2s), sAHP (small depolarization currents, sampling frequency, 10 kHz; duration, 2.5 s), IDHyperpol (depolarizing square pulses preceded by a hyperpolarizing step, sampling frequency, 10 kHz; duration, 3 s). For each cell, a holding current was applied in order to keep offset voltage at −70 mV (before liquid junction potential correction of 14 mV).

### Morphologies

The following m-types were considered in this study:

• Inhibitory m-types, DAC, descending axon cell; HAC, Horizontal axon cell; LAC, large axon cell; NGC-DA, neurogliaform cell with dense axon; NGC, neurogliaform cell; NGC-SA, neurogliaform cell with sparse axon; SAC, small axon cell; BP, bipolar cell; BTC, bitufted cell; CHC, chandelier cell; DBC, double bouquet cell; LBC, large basket cell; MC, Martinotti cell; NBC, nest basket cell; SBC, small basket cell;
• Excitatory cell m-types, IPC, inverted PC; BPC, bipolar PC; HPC, horizontal PC; TPC,A, tufted PC, late bifurcation; TPC,B, tufted PC, early bifurcation; TPC,C, tufted PC, small tuft; UPC, untufted PC; SSC, spiny stellate cell.

A total of 1,015 reconstructed rat morphologies^42^^,^^62^ (expanded dataset from Markram et al.^11^) have been curated and repaired for cut-plane missing data following the procedure outlined in Markram et al.^11^ In that study, a cloning strategy was applied by successive application of rescaling operations and jitter of section lengths and bifurcations angles. This procedure was designed so that the resulting morphologies would retain their morphological types while providing more variability across morphologies. In addition, axons and dendrites were shuffled within their original m-types to create more clones. We applied this procedure to the extended dataset of reconstructions to obtain a total of 141,733 morphologies. The relative proportions of morphological types are dictated by the expected density of morphologies of each type in SSCx (see Table S4).

### Single-cell model

#### Electrophysiological features extraction

The extraction of e-features from the voltage traces was performed using the BluePyEfe Python package.^26^ A set of e-features (as described in section “model construction and optimization”) was extracted from each patch-clamp recording for both optimizations (see list of protocols and e-features in Table 1) and validations (Table 2). The descriptions of the extracted features are summarized in Table S6. The membrane potential used as a detection threshold for the onset of an AP is −30 mV. In addition, APs are only taken into consideration if they happen during a stimulus. The e-features are averaged across cells of the same e-type. The main issue with this process is that recordings coming from different cells have different input resistances. To solve this issue we first define targets expressed as stimulus amplitudes relative to the rheobase of the cells. The rheobase of each cell is computed as the lowest stimulus amplitude inducing a spike in any of its IDrest or IDthresh recordings. The stimulus amplitudes of all recordings were normalized by the rheobase of their respective cells. Finally, e-feature vectors were averaged across cells at the target levels. For this operation, a tolerance (10% in the present study) is used as a binning width around the target stimulus amplitudes. For example, for a tolerance of 10% and a target at 150% rheobase, e-features are averaged for stimuli with amplitudes ranging from 140% to 160%. The standard deviations of the e-features at the targets are computed following the same protocol. The percentages we use as targets were chosen based on the availability of the experimental data.

### Single-neuron models and optimization

Morphologies were manually reconstructed. The axons and their branches were replaced by a synthetic axon section consisting of an AIS (60 μm) followed by a myelinated axon segment of 1,000 μm.

The constant parameters used in the e-model can be found in Table S2. The mechanisms (ion channels and calcium dynamics) are listed in Table S3. The various mechanisms added to neuron sections of the cADpyr e-type model are shown in Figure 4A and for other interneuron e-types listed in Table S5. We chose model parameters bounds for optimization based on optimization of previous models^11^ and by trial and error. The parameter ranges were not normalized. The responses of the ion channels to step voltage clamp stimuli are presented in Figure S1.

The optimization of the e-models was carried out using BluePyOpt.^18^ As an algorithm, the IBEA^17^ method was chosen, with an offspring size of 256 individuals. This multi-objective algorithm is based on the concept of indicators, which are used to evaluate the quality of solutions by comparing them with others in the population. The main idea behind IBEA is that a good solution should be better than most of the other solutions with respect to one or more objectives. As such, it tries to cover the Pareto front as well as possible. E-features extracted from the experimental data as described in the “electrophysiological features extraction” section were used to calculate the cost function. The protocols and features used for the optimizations are listed in Table 1. We calculated the e-features scores using the following formula:

(Equation 1) $$\text{efeature}\;\text{score}=\frac{\left|{e-\text{feature}}_{\text{opt}}-{\text{efeature}}_{\text{expt}}\right|}{\sigma_{\text{expt}}}$$

where e-feature_opt_ and e-feature_expt_ are the e-feature values from optimization and experiments, respectively, and $\sigma_{\text{expt}}$ is the standard deviation of the experimental e-features. Since the optimization algorithm is multi-objective, the feature scores are not combined further. To select the best solutions of the final population, the sum of all features scores was used.

During each evaluation of the model the following steps are followed:

1. Calculation of the RMP. For a set of e-model parameters obtained from the optimizer, we computed the soma RMP of the model when no stimulus is applied.
2. Calculation of the input resistance (Rin). We use a bisection search to find the holding current that brings the model to the same holding membrane potential as in the experimental Rin protocol.
3. Calculation of the threshold current. A bisection search is then also used to find the depolarizing threshold current for generating one spike in the model. If the scores for the Rin and RMP e-features are above the given limits (three standard deviations of respective mean e-feature value), the evaluation is stopped and no further protocols are applied.
4. Evaluation of the other protocols. If the model passes step 3, responses and e-features for other protocols, such as IDrest, APWaveform, and IV, are evaluated.

The optimizations for the e-types cADpyr, bAC, cACint, bNAC, cNAC, and dNAC were carried out in a single stage with the optimization algorithm running for 100 generations (with an offspring size of 256). We demonstrate the evolution of the sum of objectives score for the cADpyr L5TPC e-model during optimization (Figure S4). The evolution of optimized parameter values for the same model are depicted in Figure S5. Although we depict parameter values as normalized in these illustrations, we utilized actual values throughout the optimization process.

The models with irregular firing (bIR, cIR, bSTUT, cSTUT, and dSTUT) used a two-stage optimization approach. These e-models include the stochastic potassium channel StochKv3 to introduce stochasticity in the firing patterns. The stochastic channel can work in two modes: deterministic and non-deterministic mode. In the first stage of the two-staged optimization, all the variable e-model parameters, including the maximum conductance of the stochastic channel (in deterministic mode), were optimized. In the second stage, all the optimized parameters from the first stage are obtained and fixed except the stochastic channel conductances. The model is then optimized keeping non-deterministic mode on for this channel. The second stage of optimization was run for 100 generations with 256 offspring per generation. Both stages of optimizations follow all four steps of model evaluation as mentioned above. However, in the second stage, only one or two IDrest protocols (that were not used in the first stage) are used and features relating ISIs and burst number are evaluated. See Table 1 for protocols and features used for different e-types.

The Blue Brain 5 system (see “hardware” section) was used to run e-model optimizations. We ran numerous optimization iterations to select the final best e-models with the lowest sum of all feature scores. One such iteration for cADpyr_L5TPC, running one optimization seed using 96 computer cores, completed 100 generations of 256 offspring in about 13 h. The memory load utilization was not recorded during the optimization. We ran each e-model with 1–10 random starting seeds for optimization to generate different starting populations.

### Model validation

Somatic and dendritic L5PC validations were performed using the BluePyEfe and BluePyOpt tools. The protocols and features used for somatic validations are listed in Table 2. We selected a protocol to evaluate the cell’s subthreshold (sAHP), threshold (Ramp), and hyperpolarization properties (sAHP, IDHyperpol), including firing properties post-hyperpolarization (IDHyperpol). We extracted e-features from these protocols and compared them to empirical data, following the same method used in the single-cell optimization routine. In particular, several features were extracted in a specific manner:

• For the sAHP protocol, the sag _ ratio and sag _ amplitude were measured for the AHP that occurs after the short depolarization step.
• For the IDHyperpol protocol, the sag _ ratio and sag _ amplitude were measured during the hyperpolarizing step, while features that correspond to firing properties were measured during the depolarizing step.

For the dendritic validations, *in silico* bAP recordings were performed with maximum distances from the soma of 900 μm for apical and 150 μm for basal dendrites. Diameters of apical dendrites were measured at the midpoint from soma to the apical point, and for basal dendrites at the midpoint from soma to endpoint.

We simulated the EPSP using probabilistic AMPA and NMDA receptors with presynaptic short-term plasticity. These receptors were modeled as conductances with a double-exponential profile, following the approach of Fuhrmann et al. and Markram et al.^11^^,^^63^ EPSP amplitude attenuation ratios were measured from the resting potential to the maximum of the EPSP during transient synaptic conductance change. These EPSPs were induced at each dendritic section and measured at different locations on apical or basal dendrites. Exponential fits used the Levenberg-Marquardt algorithm.^64^

### Sensitivity analysis

The sensitivity analysis was performed for each parameter present in the e-model. Each parameter value was modified at a time, while the rest remained intact. At each step, the e-feature values were computed. These values were compared with the control e-features (with all the parameter values remaining unchanged). Each parameter was decreased by 10%, 50%, and 90%. The final sensitivity value for the parameter was computed as a slope of the curve that represents the differences in e-feature between control and modified e-models, and the slope was calculated as linear regression:

$$\text{sensitivity}\left(p_{i}\right)=\frac{\sum \left(x_{i}-\text{mean}\left(X\right)\right)\sum \left(d\left(p_{i}\right)-\text{mean}\left(d\left(p_{i}\right)\right)\right)}{\sum {\left(x_{i}-\text{mean}\left(X\right)\right)}^{2}}$$

where $d\left(p_{i}\right)$ is the difference between control values of the e-features e−features(control) and e-features obtained via modification of the *i*th parameter in the parameter set ($p_{i}$) and *X* corresponds to the percentages by which the *i*th parameter was modified; therefore, mean(X) is the average of the percentages.

### Electrical model generalization

The procedure to assign e-models to each morphology in order to create me-models is as follows. For a given cloned morphology, we selected a list of possible optimized e-models that match its me-type. Next, we evaluated the protocols used for the optimization and recorded the scores for each e-model.

We compared these scores with the scores computed on the exemplar morphology and accepted a me-model according to the following rule:

(Equation 2) $$s_{i,c}<\max\left(5,5*s_{i,e}\right)\forall i\text{,}$$

with $s_{i,e}$ the score of the exemplar morphology for a feature indexed by *i*, and $s_{i,c}$ the score of the me-model for the same feature. This procedure yielded 233,941 valid me-models, which were then used to study the generalization of the e-models and to create a circuit model. A large number of evaluations was performed in parallel using the open-source BluePyMM software.^27^

### Hardware

The code to build and validate the e-models was run on the Blue Brain 5 system,^65^ and a cluster computer consisting of hundreds of nodes with Intel Xeon processors connected the Infiniband network^66^ and a shared General Parallel File System (GPFS).^67^ Job scheduling across the system’s components was performed by the Slurm workload manager.^68^

## Data Availability

To illustrate the usage of our workflow, we prepared a set of Python notebooks: https://github.com/BlueBrain/SSCxEModelExamples.^69^ They allow the reader to run each step of the pipeline: feature extraction, optimization, validation, and generalization. The notebooks were developed for the L5PC example. The workflow encompasses several aspects that render it universally applicable. On the input side, we support numerous widely used formats, such as NWB, Igor, and axon for electrophysiological data, in addition to SWC and Neurolucida for morphologies. Our system is compatible with Neuron^30^ and Arbor^70^ simulators, and, in terms of model output, we can generate Neuron HOC models or NeuroML models. This allows for compatibility with various neurocircuit simulation tools such as BMTK^71^ and Snudda.^72^ Parts of the pipeline, including feature extraction tools such as BluePyEfel and BluePyOpt, have been successfully used in other neuroscience simulation studies,^10^ as well as for the optimization of single cells using the Allen Brain dataset.^50^ Detailed explanations of the notebooks, required packages, and instructions for running them can be found in the repository’s README file. Additionally, the repository includes continuous integration tests to guarantee the reproducibility of the results. Figure 8 visualizes the organization of the repository.

## Supplemental information

Document S1. Figures S1–S5 and Tables S1–S6

Document S2. Article plus supplemental information
