# Supplementary material for: A universal workflow for creation, validation, and generalization of detailed neuronal models
Source: Patterns (N Y). 2023 Oct 4;4(11):100855. doi: 10.1016/j.patter.2023.100855 (PMC10682753; doi:10.1016/j.patter.2023.100855)
Supplement: Document S1. Figures S1–S5 and Tables S1–S6 [file mmc1.pdf]

Patterns, Volume *4*

Supplemental information

A universal workflow for creation, validation, and generalization of detailed neuronal models

Maria Reva, Christian Rössert, Alexis Arnaudon, Tanguy Damart, Darshan Mandge, Anıl Tuncel, Srikanth Ramaswamy, Henry Markram, and Werner Van Geit

Supplementary Information

Feature ⌋ Firing Type	bAC	bNAC	bIR	bSTUT	dNAC	dSTUT	cAC	cNAC	cIR	cSTUT	cADPYR
Mean frequency (HZ)	13.30 ± 5.19	24.78 ± 9.81	12.48 ± 5.18	19.20 ± 6.15	66.17 ± 3.43	51.88 ± 7.30	16.16 ± 6.09	40.13 ± 17.37	8.81 ± 6.62	43.17 ± 19.10	19.32 ± 2.8
Number of bursts	1.15 ± 0.25	1.17 ± 0.33	1.54 ± 0.72	1.75 ± 0.77	1.05 ± 0.12	1.86 ± 1.05	1.19 ± 0.29	1.22 ± 0.56	1.79 ± 0.68	2.06 ± 0.69	1.28 ± 0.39
log(ISI slope)	0.32 ± 0.3	0.09 ± 0.12	0.37 ± 0.7	0.21 ± 0.56	-0 ± 0.05	0.04 ± 0.06	0.46 ± 0.55	0.08 ± 0.06	0.29 ± 0.28	-0 ± 0.23	0.05 ± 0.31
AP FWHM (ms)	1.09 ± 0.25	1.02 ± 0.29	1.04 ± 0.07	0.95 ± 0.22	0.80 ± 0.05	0.91 ± 0.13	1.25 ± 0.15	0.62 ± 0.03	0.67 ± 0.03	0.85 ± 0.15	1.58 ± 0.35
AP amplitude (mV)	62.65 ± 10.21	60.13 ± 8.69	59.99 ± 3.96	60.73 ± 9.20	49.25 ± 2.42	50.51 ± 6.54	67.88 ± 7.79	59.11 ± 12.96	269.55 ± 124.30	49.90 ± 8.45	94.21 ± 15.23
Input resistance (Ohm)	368.11 ± 124.34	253.30 ± 104.77	291.60 ± 117.56	198.90 ± 62.88	137.75 ± 42.50	148.65 ± 44.88	282.42 ± 114.20	215.03 ± 85.61	269.55 ± 124.30	200.47 ± 86.12	47.52 ± 15.15

Table S1: Values of the extracted e-features for 11 firing type as in Fig.2B. The values are reported as mean $\pm$ SD.

**Property**	**Value**
Resting Membrane Potential (RMP)	-80 mV
Temperature	34 °C
Specific Membrane Capacitance (cm), Soma	$1\mu F/cm^2$
Specific Membrane Capacitance (cm), Dendrites	$2\mu F/cm^2$
Specific Membrane Capacitance (cm), Myelinated Axon	$0.02\mu F/cm^2$
Axial Resistance	100 Ωcm
Na^+ Nernst Potential	50 mV
K^+ Nernst Potential	-90 mV
Initial Ca^+ Concentration	6.5x10^{-5} mM

Table S2: Constant parameters of the electrical model, related to Methods section: Single cell model (Single Neuron Models and Optimization).

**Mechanism Name**	**Parameter abbreviation**	**Reference**
**Sodium (Na) Channels**		
Transient Na	$\bar{g}$Na_T (maximum conductance)	[S1]
Persistent Na	$\bar{g}$Na_P (maximum conductance)	[S2]
**Potassium (K) Channels**		
Transient K	$\bar{g}$K_T (maximum conductance)	[S3]
Persistent K	$\bar{g}$K_P (maximum conductance)	[S3]
Kv3.1 (Shaker channels)	$\bar{g}$Kv3.1 (maximum conductance)	[S4], [S5]
Slow inactivating K	$\bar{g}$K_D (maximum conductance)	[S6]
Stochastic K	$\bar{g}$K_Stoch (maximum conductance)	[S7], [S8]
Small-conductance Ca-activated K	$\bar{g}$SK (maximum conductance)	[S9]
**Calcium (Ca) Channels**		
High-voltage-activated Ca	$\bar{g}$Ca_HVA (maximum conductance)	[S10, S11, S12]
Low-voltage-activated Ca	$\bar{g}$Ca_LVA (maximum conductance)	[S13, S14]
**Hyperpolarization-activated current**	$\bar{g}$Ih (maximum conductance)	[S15]
**Calcium Dynamics**	decay_CaDynamics (Ca decay constant), gamma_CaDynamics (% of free Ca)	[S16]

Table S3: Active mechanisms and their respective parameters used for constructing e-models, related to Methods section: Single cell model (Single cell model building workflow, Single Neuron Models and Optimization).

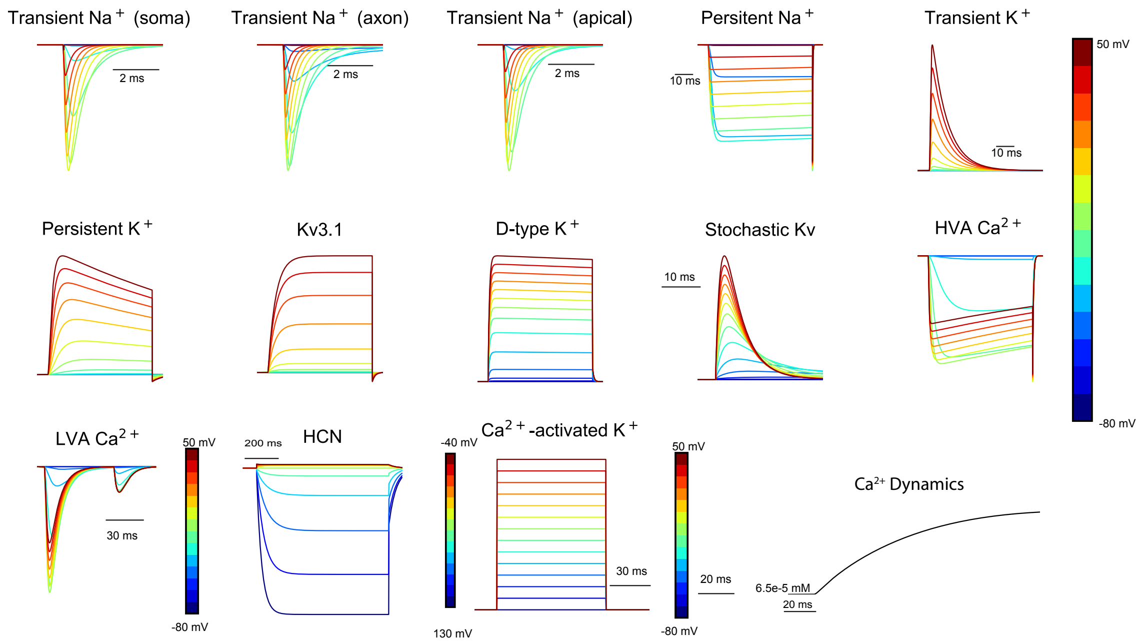

Figure S1: Ion channels currents used in cell models recorded for different voltage clamp injections. A calcium dynamics evolution with time is shown in black, see Results section (Model construction and optimizations) and Methods section: Single cell model (Single Neuron Models and Optimization).

m-type	nb. repair	nb. clone
L1_DAC	16	1680
L1_HAC	27	3645
L1_LAC	10	750
L1_NGC-DA	19	3420
L1_NGC-SA	19	1425
L1_SAC	14	1890
L23_BP	2	2478
L23_BTC	28	5612
L23_CHC	10	1600
L23_DBC	36	3960
L23_LBC	82	17220
L23_MC	29	5889
L23_NBC	36	8190
L23_NGC	8	1452
L23_SBC	44	2216
L2_IPC	4	1044
L2_TPC:A	4	1440
L2_TPC:B	33	19804
L3_TPC:A	51	18360
L3_TPC:C	11	2479
L4_BP	2	808
L4_BTC	7	1684
L4_CHC	1	328

L4_DBC	7	1478
L4_LBC	31	6510
L4_MC	17	2805
L4_NBC	25	2500
L4_NGC	1	600
L4_SBC	14	700
L4_SSC	10	2400
L4_TPC	36	25924
L4_UPC	34	16324
L5_BP	3	1148
L5_BTC	11	2200
L5_CHC	3	240
L5_DBC	9	2573
L5_LBC	21	10710
L5_MC	38	10830
L5_NBC	22	12658
L5_SBC	4	400
L5_TPC:A	64	27524
L5_TPC:B	38	8740
L5_TPC:C	30	4200
L5_UPC	27	6750
L6_BPC	28	15124
L6_BTC	1	1718
L6_CHC	1	640
L6_DBC	2	3135
L6_HPC	21	10714
L6_IPC	26	21064
L6_LBC	24	12960
L6_MC	20	9500
L6_NBC	11	9936
L6_NGC	1	788
L6_SBC	5	625
L6_TPC:A	20	8400
L6_TPC:C	18	7200
L6_UPC	17	8164

Table S4: Number of repaired and cloned morphologies used for electrical model generalization, related to Methods section: Single cell model (Morphologies).

**E-type**	Soma	AIS	Dendrites
cADpyr	CaDynamics	CaDynamics	CaDynamics
	HVA Ca	HVA Ca	HVA Ca
	LVA Ca	LVA Ca	LVA Ca
	Kv3.1	Kv3.1	Kv3.1 (Apical)
	Ca-activated K	Ca-activated K	
	Persistent K	Persistent K	
	Transient K	Transient K	
	Transient Na	Transient Na	Transient Na (Apical; Decaying)
		Persistent Na	
	HCN		HCN (Apical and Basal; Exponential increasing towards terminals)
bNAC, bAC, cNAC, cACint	CaDynamics	CaDynamics	CaDynamics
	HVA Ca	HVA Ca	HVA Ca
	LVA Ca	LVA Ca	LVA Ca
	Ca-activated K	Ca-activated K	Ca-activated K
	Transient Na	Transient Na	
	Kv3.1	Kv3.1	
	Persistent K	Persistent K	
	Transient K	Transient K	
	HCN		HCN (Exponential increasing towards terminals)
dNAC	CaDynamics	CaDynamics	CaDynamics
	HVA Ca	HVA Ca	HVA Ca
	LVA Ca	LVA Ca	LVA Ca
	Ca-activated K	Ca-activated K	Ca-activated K
	Transient Na	Transient Na	
	Kv3.1	Kv3.1	
	Persistent K	Persistent K	
	Transient K	Transient K	
	HCN		HCN (Exponential increasing towards terminals)
	D-type K	D-type K	D-type K
bIR, cIR, bSTUT,cSTUT	Passive	Passive	Passive
	CaDynamics	CaDynamics	CaDynamics
	HVA Ca	HVA Ca	HVA Ca
	LVA Ca	LVA Ca	LVA Ca
	Ca-activated K	Ca-activated K	Ca-activated K
	Transient Na	Transient Na	
	Kv3.1	Kv3.1	
	Persistent K	Persistent K	
	Transient K	Transient K	
	HCN		HCN (Exponential increasing towards terminals)
	Stochastic Kv	Stochastic Kv	Stochastic Kv
dSTUT	CaDynamics	CaDynamics	CaDynamics
	HVA Ca	HVA Ca	HVA Ca
	LVA Ca	LVA Ca	LVA Ca
	Ca-activated K	Ca-activated K	Ca-activated K
	Transient Na	Transient Na	
	Kv3.1	Kv3.1	
	Persistent K	Persistent K	
	Transient K	Transient K	
	HCN		HCN (Exponential increasing towards terminals)
	Stochastic Kv	Stochastic Kv	Stochastic Kv
	D-type K	D-type K	D-type K

Table S5: Recipe for each e-type: active parameters and their compartmental placement, related to Results section (Model construction and optimizations) and Methods section: Single cell model (Single Neuron Models and Optimization).

**eFEL Feature**	**Description**
Spikecount	Number of spikes in the trace, including outside of stimulus interval
maximum_voltage_from_voltagebase	Difference between maximum voltage during stimulus and voltage base
voltage_base	The average voltage during the last 10% of time before the stimulus.
voltage_after_stim	The mean voltage after the stimulus in (stim_end + 25%*end_period, stim_end + 75%*end_period)
AP_amplitude, APlast_amp, AP1_amp, AP2_amp	The relative height of the action potential from spike onset
AHP_depth	Relative voltage values at the first after-hyperpolarization
mean_frequency	The mean frequency of the firing rate
inv_time_to_first_spike	1.0 over time to first spike; returns 0 when no spike
time_to_last_spike	time from stimulus start to last spike
inv_first_ISI, inv_second_ISI, inv_third_ISI, inv_fourth_ISI, inv_fifth_ISI, inv_last_ISI	1.0 over first/second/third/fourth/fith/last ISI; returns 0 when no ISI
AP_duration_half_width	Width of spike at half spike amplitude
ohmic_input_resistance_vb_ssse	The ratio between the voltage deflection (between voltage base and steady-state voltage at stimend) and stimulus current
voltage_deflection	The voltage deflection between voltage base and steady-state voltage at stimend
voltage_deflection_begin	The voltage deflection between voltage base and steady-state voltage soon after stimulation start.
decay_time_constant_after_stim	The decay time constant of the voltage right after the stimulus
holding_current	holding current injected to stabilize resting membrane potential
threshold_current	minimum current required to generate an AP found by bisection search
AHP_depth_abs	Absolute voltage values at the first after-hyperpolarization
burst_number	The number of bursts
ISI_CV	The coefficient of variation of the ISIs
time_to_first_spike	Time from the start of the stimulus to the maximum of the first peak
time_to_second_spike	Time from the start of the stimulus to the maximum of the second peak

Table S6: Descriptions of the eFEL feature used for optimizations and validations of e-models, related to Fig.3 and Methods section: Single cell model (Electrophysiological features extraction) More details for the features can be found at https://efel.readthedocs.io/en/latest/eFeatures.html.

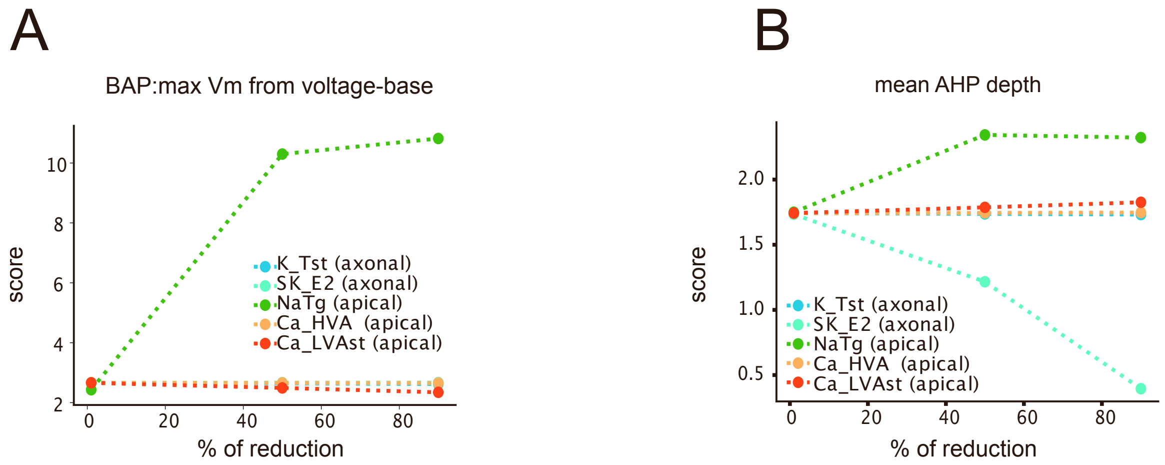

Figure S2: Examples of sensitivity analysis in L5PC models for the several parameters (K_Tst(axonal): blue, SK_E2(axonal): light blue, NaTg(apical): green, Ca_HVA(apical): orange, Ca_LVAst(apical): red), x-axis: percentage by which the parameter was reduced; y-axis: value of the e-feature score. A. Sensitivity analysis for the e-feature representing maximum voltage of the bAP. B. Sensitivity analysis for the mean AHP depth feature. Related to Results section (Validation and analysis of the detailed neuronal model) and Fig.6A.

Comparison of generalization results between current and previous edition of the L5PC e-models

To perform generalization comparison we used the same set of e-features and same morphologies for two e-models: optimized in this work and previously reported L5PC e-model [S17]. As a metric for this comparison we report the fraction of passed morphologies for each version of the e-models Fig.S3.

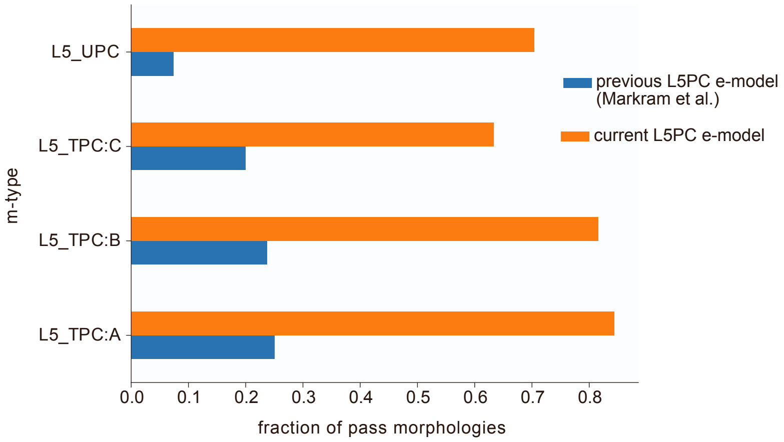

Figure S3: Comparison of generalization results between L5PC e-models from the current work (orange) and previously released e-model (blue) [S17]. Generalization was run for four morphological m-types of L5PC.

The number of e-features computed in Markram et al. [S17] were smaller, without automatic holding and current threshold detection, and show a worse generalization ability on the morphologies used in this paper. Notice that these two e-models were trained on different exemplar morphologies, both present in the current set of morphologies.

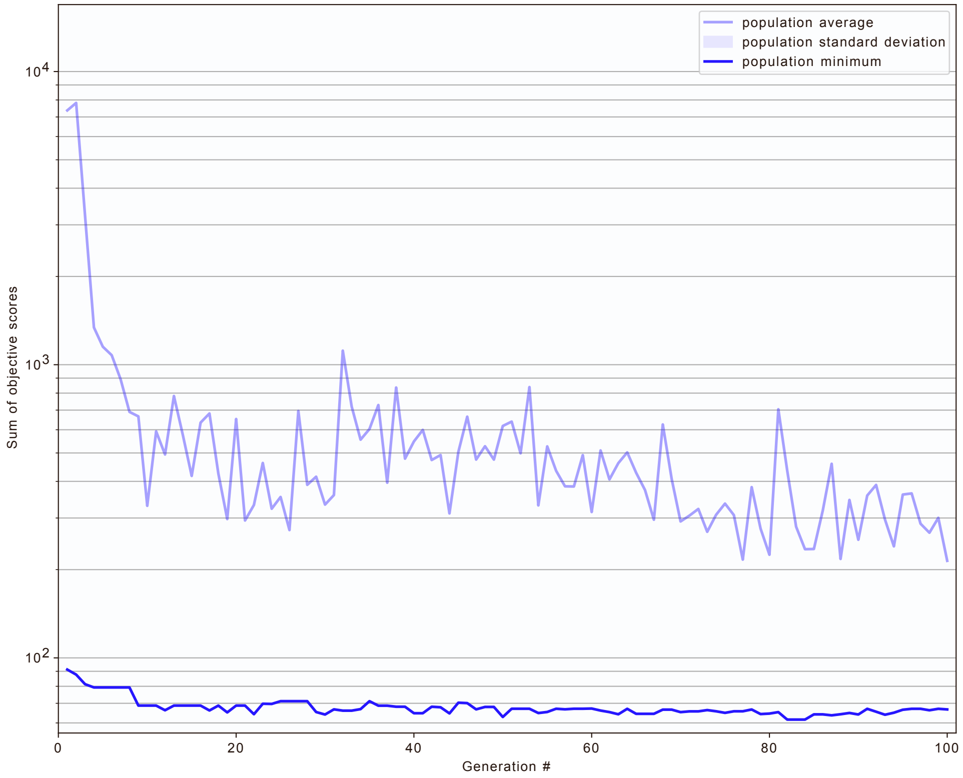

Figure S4: Optimization score evolution for cADpyr_L5TPC model during optimization: Minimum and average optimization score (sum of objectives) among offspring population for different generations, related to Methods section: Single cell model (Single Neuron Models and Optimization).

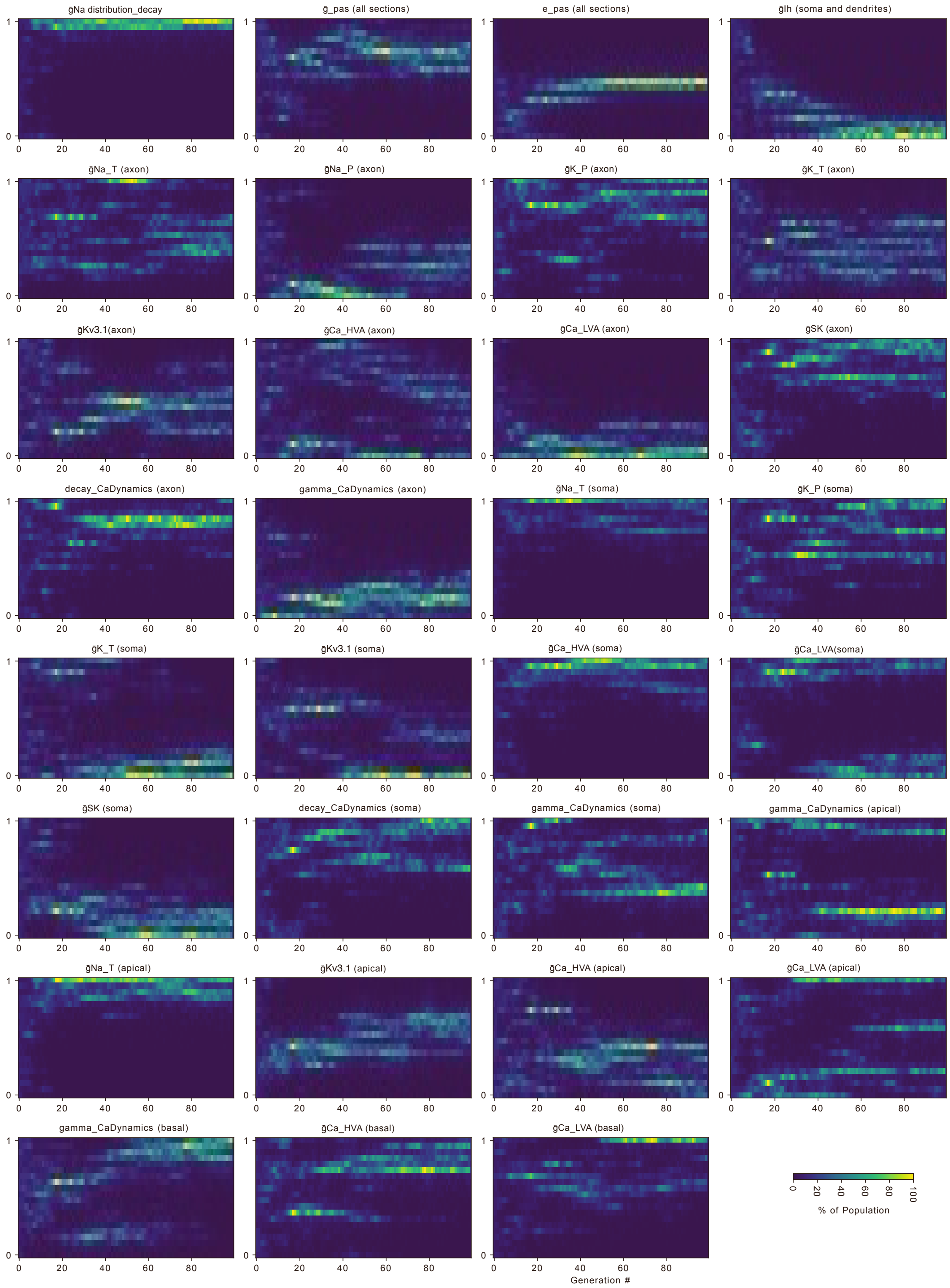

Figure S5: Parameter value evolution for cADpyr_L5TPC model during optimization: The plot shows normalized model parameter values (on y-axis) across optimization generations. The color represents the % of offspring population for a given parameter value. Here, BluePyOpt's IBEA algorithm is used to run the model for 100 generations each with an offspring of 256 individuals. Related to Methods section: Single cell model (Single Neuron Models and Optimization).

References

[S1] Costa M Colbert and Enhui Pan. "Ion channel properties underlying axonal action potential initiation in pyramidal neurons". In: *Nature neuroscience* 5.6 (2002), pp. 533–538. DOI: 10.3410/f.1007335.92105.

[S2] Jacopo Magistretti and Angel Alonso. "Biophysical properties and slow voltage-dependent inactivation of a sustained sodium current in entorhinal cortex layer-II principal neurons: a whole-cell and single-channel study". In: *The Journal of general physiology* 114.4 (1999), pp. 491–509. DOI: 10.1085/jgp.114.4.491.

[S3] Alon Korngreen and Bert Sakmann. "Voltage-gated K+ channels in layer 5 neocortical pyramidal neurones from young rats: subtypes and gradients". In: *The Journal of physiology* 525.3 (2000), pp. 621–639. DOI: 10.1111/j.1469-7793.2000.00621.x.

[S4] Jens Rettig, Frank Wunder, Martin Stocker, Ralf Lichtinghagen, Frank Mastiaux, Synnöve Beckh, W Kues, Paola Pedarzani, Klaus H Schröter, and Johann P Ruppersberg. "Characterization of a Shaw-related potassium channel family in rat brain." In: *The EMBO Journal* 11.7 (1992), pp. 2473–2486. DOI: 10.1002/j.1460-2075.1992.tb05312.x.

[S5] A Grupe, KH Schröter, JP Ruppersberg, M Stocker, Th Drewes, S Beckh, and O Pongs. "Cloning and expression of a human voltage-gated potassium channel. A novel member of the RCK potassium channel family." In: *The EMBO journal* 9.6 (1990), pp. 1749–1756. DOI: 10.1002/j.1460-2075.1990.tb08299.x.

[S6] Yousheng Shu, Yuguo Yu, Jing Yang, and David A McCormick. "Selective control of cortical axonal spikes by a slowly inactivating K+ current". In: *Proceedings of the National Academy of Sciences* 104.27 (2007), pp. 11453–11458. DOI: 10.1073/pnas.0702041104.

[S7] Kamran Diba, Christof Koch, and Idan Segev. "Spike propagation in dendrites with stochastic ion channels". In: *Journal of computational neuroscience* 20.1 (2006), pp. 77–84. DOI: 10.1007/s10870-006-4770-0.

[S8] Philipe RF Mendonça, Mariana Vargas-Caballero, Ferenc Erdélyi, Gábor Szabó, Ole Paulsen, and Hugh PC Robinson. "Stochastic and deterministic dynamics of intrinsically irregular firing in cortical inhibitory interneurons". In: *Elife* 5 (2016), e16475. DOI: 10.7554/elife.16475.026.

[S9] M Köhler, B Hirschberg, CT Bond, John Mark Kinzie, NV Marrion, James Maylie, and JP Adelman. "Small-conductance, calcium-activated potassium channels from mammalian brain". In: *Science* 273.5282 (1996), pp. 1709–1714. DOI: 10.1126/science.273.5282.1709.

[S10] I Reuveni, A Friedman, Y Amitai, and Michael J Gutnick. "Stepwise repolarization from Ca2+ plateaus in neocortical pyramidal cells: evidence for nonhomogeneous distribution of HVA Ca2+ channels in dendrites". In: *Journal of Neuroscience* 13.11 (1993), pp. 4609–4621. DOI: 10.1523/jneurosci.13-11-04609.1993.

[S11] RJ Sayer, PC Schwindt, and WE Crill. "High-and low-threshold calcium currents in neurons acutely isolated from rat sensorimotor cortex". In: *Neuroscience letters* 120.2 (1990), pp. 175–178. DOI: 10.1016/0304-3940(90)90031-4.

[S12] Marc A Dichter and Cristina Zona. "Calcium currents in cultured rat cortical neurons". In: *Brain research* 492.1-2 (1989), pp. 219–229. DOI: 10.1016/0006-8993(89)90904-99.

[S13] Robert B Avery and Daniel Johnston. "Multiple channel types contribute to the low-voltage-activated calcium current in hippocampal CA3 pyramidal neurons". In: *Journal of Neuroscience* 16.18 (1996), pp. 5567–5582. DOI: 10.1523/jneurosci.16-18-05567.1996.

[S14] AD Randall and RW Tsien. “Contrasting biophysical and pharmacological properties of T-type and R-type calcium channels”. In: *Neuropharmacology* 36.7 (1997), pp. 879–893. DOI: 10.1016/s0028-3908(97)00086-5.

[S15] Maarten HP Kole, Stefan Hallermann, and Greg J Stuart. “Single Ih channels in pyramidal neuron dendrites: properties, distribution, and impact on action potential output”. In: *Journal of Neuroscience* 26.6 (2006), pp. 1677–1687. DOI: 10.1523/jneurosci.3664-05.2006.

[S16] Alain Destexhe, Diego Contreras, Terrence J Sejnowski, and Mircea Steriade. “A model of spindle rhythmicity in the isolated thalamic reticular nucleus”. In: *Journal of neurophysiology* 72.2 (1994), pp. 803–818. DOI: 10.1152/jn.1994.72.2.803.

[S17] Henry Markram, Eilif Muller, Srikanth Ramaswamy, Michael W Reimann, Marwan Abdellah, Carlos Aguado Sanchez, Anastasia Ailamaki, Lidia Alonso-Nanclares, Nicolas Antille, Selim Arsever, et al. “Reconstruction and simulation of neocortical microcircuitry”. In: *Cell* 163.2 (2015), pp. 456–492. DOI: https://doi.org/10.1016/j.cell.2015.09.029.
